# *Paridris* Kieffer of the New World (Hymenoptera, Platygastroidea, Platygastridae)

**DOI:** 10.3897/zookeys.233.3455

**Published:** 2012-10-26

**Authors:** Elijah J. Talamas, Lubomír Masner, Norman F. Johnson

**Affiliations:** 1 Department of Entomology, The Ohio State University, 1315 Kinnear Road, Columbus, Ohio 43212, U.S.A.; 2 Agriculture and Agri-Food Canada, K.W. Neatby Building, Ottawa, Ontario K1A 0C6, Canada; 3 Department of Evolution, Ecology and Organismal Biology, The Ohio State University, 1315 Kinnear Road, Columbus, Ohio 43212, U.S.A.

**Keywords:** Egg-parasitoid, key, species description, revision, lectotype designation

## Abstract

*Paridris* in the New World is revised (Hymenoptera: Platygastridae). Fifteen species are described, of which 13 are new. *Paridris aenea* (Ashmead)(Mexico (Tamaulipas) and West Indies south to Bolivia and southern Brazil (Rio de Janeiro state)), *Paridris armata* Talamas, **sp. n.** (Venezuela), *Paridris convexa* Talamas, **sp. n.** (Costa Rica, Panama), *Paridris dnophos* Talamas, **sp. n.** (Mexico (Vera Cruz) south to Bolivia and central Brazil (Goiás)), *Paridris gongylos* Talamas & Masner, **sp. n.** (United States: Appalachian Mountains of Virginia, Tennessee, South Carolina), *Paridris gorn* Talamas & Masner, **sp. n.** (United States: Ohio south to Alabama, Georgia), *Paridris invicta* Talamas & Masner, **sp. n.** (Brazil: São Paulo), *Paridris isabelicae* Talamas & Masner, **sp. n.** (Cuba, Dominican Republic), *Paridris lemete* Talamas & Masner, **sp. n.** (Puerto Rico), *Paridris minor* Talamas, **sp. n.** (Cuba), *Paridris nayakorum* Talamas, **sp. n.** (Costa Rica), *Paridris pallipes* (Ashmead)(southeastern Canada, United States south to Costa Rica, also Brazil (São Paulo), *Paridris psydrax* Talamas & Masner, **sp. n.** (Argentina, Mexico, Paraguay, United States, Venezuela), *Paridris saurotos* Talamas, **sp. n.** (Jamaica), *Paridris soucouyant* Talamas & Masner, **sp. n.** (Colombia, Trinidad and Tobago, Venezuela). *Paridris brevipennis* Fouts, *Paridris laeviceps* (Ashmead), and *Paridris nigricornis* (Fouts) are treated as junior synonyms of *Paridris pallipes*; *Paridris opaca* is transferred to *Probaryconus*. Lectotypes are designated for *Idris aenea* Ashmead and *Caloteleia aenea* Ashmead.

## Introduction

J.J. Kieffer described the genus *Paridris* in 1908 to accommodate misinterpretations of Foerster’s (1856) genus *Idris*. Hetransferred three species to the new name: *Idris laeviceps* Ashmead, *Idris aenea* Ashmead and *Idris nigricornis* Brues, with *Idris laeviceps* selected as the type species of the new genus. One additional species, *Paridris brevipennis* Fouts, recorded as an egg parasitoid of the cricket *Gryllus pennsylvanicus* Burmeister (Masner and Muesebeck 1968), was described in 1920, and Masner (1976) transferred *Paridris opaca* (Kieffer) and *Paridris pallipes* (Ashmead) into the genus from *Paranteris* and *Thoron*, respectively.

Despite the fact that the genus was originally based on species of the Western Hemisphere, subsequent taxonomy of the genus was almost exclusively based on Old World species. Taxonomic circumscription of *Paridris* has required assessment on a world scale because of its polytypic morphology, which is perhaps most apparent among the New World species. Of the 13 new species described in this paper, 7 are morphologically close to *Paridris pallipes*, whereas the majority of the world species bear little obvious relation to the type species of the genus. The key to separate *Paridris* from *Probaryconus* and *Anteris* (Talamas et al. 2011) included specimens from the New World because it is here that *Paridris* resembles these genera most. Here we expand our study of New World *Paridris* to the species level as part of an ongoing treatment of the genus worldwide.

The gender of the name *Paridris* has been a point of confusion in previous literature, some of it of our own creation. Kieffer (1908) used the adjectival epithet “*aenea*” when transferring *Idris aenea* at the time he erected *Paridris*, thus indicating that the gender of *Paridris* is feminine. Masner (1976), Galloway and Austin (1984), Mani and Sharma (1982), Kononova and Petrov (2000), Lê (2000), Kononova and Kozlov (2008), Rajmohana and Bijoy (2011), and Talamas et al. (2011) treated *Paridris* as masculine. According to Article 30.1.4.2 of the Code, generic names must be treated as feminine names if they are treated as feminine in combination with an adjectival species-group name at the time they are established. We now treat the gender of *Paridris* accordingly and extend our thanks to David Notton (BMNH) for his detailed analysis of the matter and bringing it to our attention while reviewing our manuscript. Species epithets previously treated as masculine are as follows: *Paridris bispinosa* (Masner), *Paridris fera* Talamas, *Paridris gloria* Kononova, *Paridris pachmarhica* (Sharma), *Paridris parvoculata* Galloway, *Paridris rugulosa* Talamas, *Paridris spinosa* Rajmohana, *Paridris stena* Kononova & Petrov, and *Paridris verrucosa* Talamas.

This work is conducted as part of the Platygastroidea Planetary Biodiversity Inventory and represents a step toward a species-level revision of the Scelionini sensu lato. The contributions of the authors are as follows: E.J. Talamas: collection of specimens, character definition, species concept development, imaging, key development, manuscript preparation; L. Masner: collection and aggregation of specimens, species concept development, manuscript preparation; N.F. Johnson: software and database development, manuscript preparation.

## Materials and methods

**Specimens:** This work is based upon specimens deposited in the following collections, with abbreviations used in the text: AMNH, American Museum of Natural History, New York, USA^1^; BMNH, Natural History Museum, London, England^2^; CASC, California Academy of Sciences, San Francisco, CA^3^; CNCI, Canadian National Collection of Insects, Ottawa, Canada^4^; IAVH, Colección de Artrópodos, Instituto Alexander von Humboldt, Villa de Leyva, Colombia^5^; INBC, Instituto Nacional de Biodiversidad, Santo Domingo de Heredia, Costa Rica^6^; INHS, Illinois Natural History Survey, Champaign, Illinois, USA^7^; LACM, Natural History Museum of Los Angeles County, Los Angeles, California, USA^8^; MCZ, Harvard University Museum of Comparative Zoology, Cambridge, Massachusetts, USA^9^; MEMU, Mississippi State University, Mississippi State, MS^10^; MZLU, Lund Museum of Zoology, Lund University, Lund, Sweden^11^; MZSP, Museu de Zoologia da Universidade de São Paulo, São Paulo, Brazil^12^; OSUC, C.A. Triplehorn Insect Collection, Columbus, OH^13^; UCDC, R. M. Bohart Museum of Entomology, Davis, CA^14^; UCMC, University of Colorado Museum of Natural History, Boulder, Colorado^15^; USNM, Smithsonian National Museum of Natural History, Washington DC, USA^16^.

**Morphology:** Abbreviations and morphological terms used in text: A1, A2, ... A12: antennomere 1, 2, ... 12; claval formula: distribution of the multiporous basiconic sensilla on the underside of apical antennomeres of the female, with the antennomere interval specified followed by the number of sensilla per segment (Bin 1981); palpal formula: number of maxillary and labial palpal segments, respectively; S1, S2, ... S6: metasomal mediosternite 1, 2, ... 6; T1, T2, ... T7: metasomal mediotergite 1, 2, ... 7.; posterior vertex: area between the posterior ocelli and the occipital carina. Morphological terminology largely follows Mikó et al. 2007; the following are illustrated and labeled to facilitate their use.

anterior propodeal projection (app: Fig. 12)

lateral propodeal area (lpa: Figs 11, 13)

lateral propodeal carina (lpc: Figs 11–13)

occipital carina (occ: Figs 9, 10, 17)

plical carina (plc: Figs 11, 13)

plical area (pla: Figs 12, 13)

pronotal cervical sulcus (prcs: Fig. 29)

pronotal suprahumeral sulcus (pss: Fig. 29)

postacetabular sulcus (ats: Fig. 10)

postgena (pg: Figs 8–10)

prespiracular propodeal area (pspp: Figs 11–13)

transverse carina of T2 (trc; Fig. 70)

Morphological terms used in this revision were matched to the Hymenoptera Anatomy Ontology (HAO, Yoder et al. 2010) (Appendix 1). Identifiers (URIs) in the format http://purl.obolibrary.org/obo/HAO_XXXXXXX represent anatomical concepts in HAO version http://purl.obolibrary.org/obo/hao/2011-05-18/hao.owl. They are provided to enable readers to confirm their understanding of the anatomical structures being referenced. To find out more about a given structure, including, images, references, and other metadata, use the identifier as a web-link, or use the HAO:XXXXXXX (note colon replaces underscore) as a search term at http://glossary.hymao.org.

The description of surface sculpture is presented in two formats. Areas of the exoskeleton in which the sculptural elements are inseparable are described simply as “sculpture”. For areas in which the sculptural elements vary independently, sculpture is divided into three categories: punctation: round depressions associated with setae; macrosculpture: raised or sunken patterns of texture that are oriented linearly or radially with respect to punctation or the axes of the body; microsculpture: unoriented, very fine wrinkles or pustulations that occur on, in, or between elements of macrosculpture and punctation.

**Information management:** The locality data reported for primary types are not a literal transcription of the labels: some abbreviations are expanded; additional data from the collectors are also included. The holotypes should be unambiguously identifiable by means of the unique identifier or the red holotype label. The numbers prefixed with “OSUC ” and “CASENT ” are unique identifiers for the individual specimens (note the blank space after the acronyms). Details on the data associated with these specimens may be accessed at the following link, purl.oclc.org/NET/hymenoptera/hol, and entering the identifier in the form. This monograph also features simultaneous publication and distribution of taxonomic and occurrence records through the Global Biodiversity Information Facility (GBIF) using DarwinCore Archives. All new species have been prospectively registered with Zoobank (Polaszek et al. 2005) and other taxonomic names have been retrospectively registered therein. All names are also registered in the Hymenoptera Name Server (http://hns.osu.edu/). Life sciences identifiers, lsids, may be resolved at the URLs specified in the footnotes or at lsid.tdwg.org.

**Cybertools:** The species descriptions are generated by a database application, vSysLab (purl.oclc.org/NET/hymenoptera/vSysLab), designed to facilitate the generation of taxon by character data matrices, to integrate these with the existing taxonomic and specimen-level database, and to export the data both as text and as input files for other applications. The output is in the format of “Character: Character state(s).” Polymorphic characters are indicated by semicolon-separated character states.

**Imaging:** Images were produced using Combine ZP and AutoMontage extended-focus software. The individual images are archived at the image database at The Ohio State University (purl.oclc.org/NET/hymenoptera/specimage) and with MorphBank (www.morphbank.net). The latter also contains collections of images organized by plate.

**Species concept:** For the purpose of this revision, species are defined as taxa diagnosable by putative autapomorphies or a unique combination of fixed character states.

**Identification keys** (a Lucid key is included as a supplementary file and is also available at http://hymfiles.biosci.ohio-state.edu/keys/1/)

## Key to Females (unknown for *Paridris armata*, *Paridris invicta*, *Paridris gongylos*)

**Table:** 

1	Genal striae weakly developed, rarely reaching ventral margin of eye (Figs 26, 29, 30, 37); plical carina absent and lateral propodeal area indistinguishable from plical area (Figs 12, 66); occipital carina not extending below foramen magnum (Figs 8, 10); T2 without transverse carina (Figs 12, 27, 31, 35)	2
–	Genal striae pronounced, extending above ventral margin of eye (Figs 14, 18, 19, 38); lateral propodeal area differentiated from plical area by distinct plical carina (Figs 11, 13); occipital carina reaching base of mandible (Fig. 9) or antecostal sulcus of T2 bordered posteriorly by transverse carina (Figs 15, 20, 39, 41)	8
2	Antecostal sulcus of T2 comprised of deep cells (Figs 12, 31, 60); posterior margin of sulcus strongly convex (Figs 31, 60); lateral T3 with longitudinal line of setae (Figs 30, 59); T3–T5 without macrosculpture (Figs 31, 60)	3
–	Antecostal sulcus of T2 present as a constriction, without deep cells (Figs 27, 35, 47, 64); posterior margin of sulcus weakly convex (Figs 27, 35, 47, 64); lateral T3 without longitudinal line of setae (Figs 26, 34, 63, 71); macrosculpture of T3–T5 variable	4
3	A8 with 2 basiconic sensilla (Fig. 6); metascutellum obscured by horn of T1 (Fig. 60); T1 without longitudinal striae (Fig. 60)	*Paridris nayakorum* Talamas, sp. n.
–	A8 with 1 basiconic sensillum (as in Fig. 7); metascutellum visible (Figs 12, 31); T1 longitudinally striate (Figs 12, 31)	*Paridris dnophos* Talamas, sp. n.
4	T6 evenly convex, usually smooth medially (Figs 56, 64, 83, 84); ventral metapleural area setose and punctate throughout (Fig. 63)	5
–	T6 apically constricted, densely and finely punctate throughout (Figs 81, 82); setation and sculpture of ventral metapleural area variable	6
5	Notaulus percurrent, reaching mesoscutal suprahumeral sulcus as a smooth furrow (Fig. 55)	*Paridris minor* Talamas, sp. n.
–	Notaulus abbreviate, or at most reaching mesoscutal suprahumeral sulcus as a line of punctures (Fig. 64)	*Paridris pallipes* (Ashmead)
6	Posterior surface of horn on T1 entirely smooth (Figs 46, 47)	*Paridris isabelicae* Talamas & Masner, sp. n.
–	Posterior surface of horn on T1 not entirely smooth (Figs 26, 71)	7
7	Head with reticulate microfissures throughout (Figs 26, 27, 29); posterior surface of horn on T1 with transverse ridge (Figs 26, 27)	*Paridris convexa* Talamas, sp. n.
–	Head with reticulate microfissures limited to patches between median and lateral ocelli, on temples, on anterodorsal margin of eye or directly posterior to lateral ocellus (Figs 71, 72); posterior surface of horn on T1 with posteriorly directed spine (Figs 71, 72)	*Paridris saurotos* Talamas, sp. n.
8	Metascutellum obscured by large horn of T1 (Fig. 68, 70); posterior head and anterior mesosoma with dense pustulate microsculpture (Figs 67, 68); mandible unidentate or with ventral tooth minute (Fig. 69); length of T6 greater than width along anterior margin (Fig. 80)	*Paridris psydrax* Talamas & Masner, sp. n.
–	Metascutellum visible (Figs 13, 15, 39, 76); head and anterior mesosoma without pustulate microsculpture (Figs 15, 19, 39, 76); mandible tridentate, medial tooth the smallest (Fig. 17); length of T6 less than or equal to width along anterior margin (Fig. 79)	9
9	Metascutellum bispinose (Figs 39, 41, 76, 78); sculpture of posterior head irregularly rugulose (Figs 39, 76)	10
–	Posterior margin of metascutellum straight or convex, rarely emarginate; if emarginate then irregularly so and without lateral points (Figs 15, 51, 53); sculpture of posterior head variable	11
10	Horn of T1 with longitudinal carina along its dorsal crest (Fig. 78); T4–T5 strigose to rugulose laterally (Fig. 76)	*Paridris soucouyant* Talamas & Masner, sp. n.
–	Horn of T1 simple, sometimes with median, longitudinal row of shallow punctures (Figs 38, 39); T4–T5 without macrosculpture (Fig. 39)	*Paridris gorn* Talamas & Masner, sp. n.
11	A8 with single basiconic sensillum (Fig. 7); horn of T1 with strong, posteriorly directed spine, without carinate crest (Fig. 14); micropunctation often present on horn (Fig. 13)	*Paridris aenea* (Ashmead)
–	A8 with 2 basiconic sensilla (as in Fig. 6); horn of T1 sometimes with weak spine directed posteriorly, with carinate crest (Figs 50, 51, 53); micropunctation not present on horn (Fig. 53)	*Paridris lemete* Talamas & Masner, sp. n.

## Key to Males (unknown for *Paridris nayakorum*)

**Table:** 

1	Genal striae weakly developed, rarely reaching ventral margin of eye (Figs 26, 29, 30, 37); plical carina absent and lateral propodeal area indistinguishable from plical area (Figs 12, 66); occipital carina not extending below foramen magnum (Figs 8, 10); T2 without transverse carina (Figs 12, 27, 31, 35)	6
–	Genal striae pronounced, extending above ventral margin of eye (Figs 14, 18, 19, 38); lateral propodeal area differentiated from plical area by distinct plical carina (Figs 11, 13); occipital carina reaching base of mandible (Fig. 9) or antecostal sulcus of T2 bordered posteriorly by transverse carina (Figs 15, 20, 39, 41)	2
2	Posterior margin of metascutellum emarginate (Figs 22, 41, 76, 78)	3
–	Posterior margin of metascutellum straight or convex (Figs 13, 53)	5
3	Clypeus smooth along ventral margin and narrower than torular space (Fig. 24); posterior margin of antecostal sulcus on T2 convex and without transverse carina (Fig. 23); notaulus absent in anterior half of mesoscutum and poorly defined posteriorly (Fig. 23)	*Paridris armata* Talamas, sp. n.
–	Clypeus serrate along ventral margin and wider than torular space (Fig. 40); posterior margin of antecostal sulcus on T2 straight, bordered posteriorly by transverse carina (Figs 39, 41, 76); notaulus percurrent or reaching mesoscutal suprahumeral sulcus as a line of punctures	4
4	T4–T5 strigose to rugulose laterally (Fig. 76)	*Paridris soucouyant* Talamas & Masner, sp. n.
–	T4–T5 without macrosculpture (Fig. 39)	*Paridris gorn* Talamas & Masner, sp. n.
5	Lateral propodeal area discontiguous with prespiracular propodeal area (Fig. 11); femora enlarged (Fig. 42); gena with dense long setae (Fig. 44)	*Paridris invicta* Talamas & Masner, sp. n.
–	Lateral propodeal area contiguous with prespiracular propodeal area (Figs 13, 53, 70); femora not obviously enlarged; setation of gena either not dense or long	6
6	A6–A11 spherical in shape (Fig. 5); head, anterior mesoscutum and metasoma covered with dense pustulate microsculpture (Fig. 68)	*Paridris psydrax* Talamas & Masner, sp. n.
–	A6–A11 longer than wide (Fig. 4); head, mesoscutum and metasoma without dense microsculpture (Figs 15, 51)	7
7	Mesopleuron below femoral depression coarsely punctate rugose to areolate (Fig. 85); postmarginal vein about half as long as stigmal vein (Fig. 87)	*Paridris aenea* (Ashmead)
–	Mesopleuron below femoral depression mostly smooth with sparse large punctures (Fig. 86); postmarginal vein as long as stigmal vein (Fig. 88)	*Paridris lemete* Talamas & Masner, sp. n.
8	Length of flagellomeres (A6–A11) greater than 3 times width (Fig. 1)	9
–	Length of flagellomeres (A6–A11) less than 3 times width (Fig. 2)	11
9	Reticulate microfissures present throughout posterior vertex and temples (Figs 26, 27)	*Paridris convexa* Talamas, sp. n.
–	Posterior vertex entirely smooth (Figs 46, 47, 71, 72), microsculpture sometimes present in small patch on temples and between median and lateral ocelli	10
10	Medial mesoscutum with dense to moderately dense setigerous punctation throughout (Fig. 72); medial S2 smooth (Fig. 74)	*Paridris saurotos* Talamas, sp. n.
–	Medial mesoscutum mostly smooth and glabrous with very sparse setigerous punctures (Fig. 47); medial S2 longitudinally striate (Fig. 49)	*Paridris isabelicae* Talamas & Masner, sp. n.
11	Antecostal sulcus of T2 comprised of deep cells (Figs 12, 31); posterior margin of sulcus strongly convex (Fig. 31); lateral T3 longitudinal line of setae (Fig. 30); T3–T5 without macrosculpture (Fig. 31)	*Paridris dnophos* Talamas, sp. n.
–	Antecostal sulcus of T2 present as a constriction, without deep cells (Figs 35, 56, 64); posterior margin of sulcus weakly convex (Figs 35, 56, 64); lateral T3 without longitudinal line of setae (Fig. 34); macrosculpture of T3–T5 variable	12
12	Occipital carina crenulate anteriorly (Fig. 35)	*Paridris gongylos* Talamas & Masner, sp. n.
–	Occipital carina simple (Fig. 55, as in Fig. 72)	13
13	Notaulus percurrent, reaching mesoscutal suprahumeral sulcus as a smooth furrow (Fig. 55)	*Paridris minor* Talamas, sp. n.
–	Notaulus abbreviate, or at most reaching mesoscutal suprahumeral sulcus as a line of punctures (Fig. 64)	*Paridris pallipes* (Ashmead)

## Taxonomy

### Paridris aenea

(Ashmead)

urn:lsid:zoobank.org:act:8709F7AA-46A7-4D34-98F8-9671D6539ABF

urn:lsid:biosci.ohio-state.edu:osuc_concepts:5062

http://species-id.net/wiki/Paridris_aenea

Figures 4 13 [Fig F3] 20 85, 87 Morphbank^17^

Idris aenea Ashmead, 1894: 231 (original description); Ashmead 1900: 328 (distribution).
Paridris aenea (Ashmead): Kieffer 1908: 123 (generic transfer); Kieffer 1926: 421, 423 (description, keyed); Masner 1976: 36 (type information, description, emendation).
Caloteleia aenea Ashmead, 1894: 218, 219 (original description, keyed) syn. n.; Ashmead 1900: 327 (distribution).
Ceratoteleia aenea (Ashmead): Kieffer 1908: 121 (generic transfer).
Oxyteleia aenea (Ashmead): Kieffer 1926: 516, 517 (generic transfer, description, keyed). urn:lsid:zoobank:act:39C31284-74AC-4431-B2AF-0771E8E9603C urn:lsid:biosci.ohio-state.edu:osuc_concepts:9489

#### Description.

Female body length: 1.73–2.66 mm (n=20). Male body length: 1.38-2.54 mm (n=20).

Number of basiconic sensilla on A8: 1.

Color of head: brown; black. Distal margin of clypeus: serrate. Width of clypeus: wider than interantennal process. Lateral corner of clypeus: projecting into acute angle. Development of interantennal process ventrally: not reaching clypeus. Number of mandibular teeth: three. Length of mediofacial striae: not extending above midpoint of eye; extending to dorsal frons. Shape of gena in dorsal view: moderately receding behind compound eye. Striae on gena: pronounced. Length of striae on gena: extending above ventral margin of eye. Distribution of microsculpture on head: absent. Length of OOL: greater than 2 ocellar diameters; less than 2 ocellar diameters. Occipital carina above foramen magnum: present. Anterior margin of occipital carina: comprised of small to miniscule cells. Setation of postgena: sparse. Ventral extent of occipital carina: extending to base of mandible.

Color of mesosoma: yellowish brown to black; reddish brown. Dorsal half of pronotal cervical sulcus: present as line of small to minute cells; present as smooth furrow. Ventral half of pronotal cervical sulcus: present as line of small to minute cells. Transverse pronotal carina: present in posterior half of pronotum; present in posterodorsal corner of pronotum. Shape of pronotal shoulder in dorsal view: narrow and striplike. Form of pronotal suprahumeral sulcus: areolate. Macrosculpture of anterior medial mesoscutum: absent. Density of punctation on anterior medial mesoscutum: dense along mesoscutal suprahumeral sulcus, otherwise sparse; dense throughout. Reticulate microfissures on anterior half of medial mesoscutum: absent. Pustulate microsculpture on anterior mesoscutum: absent. Density of punctation on posterior medial mesoscutum: sparse. Notaulus: percurrent, reaching suprahumeral sulcus as a smooth furrow; percurrent, reaching suprahumeral sulcus as a line of punctures. Orientation of notauli: parallel. Shape of notaulus at posterior apex: ovoid. Macrosculpture of mesoscutellum: punctate rugose along margins, smooth medially. Postacetabular sulcus: crenulate. Mesopleural carina: present, complete. Punctures on posterodorsal mesepimeral area: very fine; absent; large. Sculpture of mesopleuron anteroventral to femoral depression: areolate to punctate rugose throughout; densely punctate on lateral surface, smooth on ventral surface. Sculpture of posterior mesepimeral area: smooth. Form of metascutellum in female: transverse punctate rugulose lamella, posterior margin approximately straight. Form of metascutellum in male: transverse punctate rugulose lamella, posterior margin approximately straight. Paracoxal and metapleural sulci: separate. Posterior margin of metapleuron below propodeal spiracle: straight to moderately convex. Setation between metapleural triangle and metapleural sulcus: absent. Sculpture between metapleural triangle and metapleural sulcus: smooth; punctate; punctate rugose; faintly rugulose. Sculpture of metapleural triangle: punctate rugose. Setation of metapleural triangle: sparse. Anterior propodeal projection: absent. Setation of metasomal depression: absent. Lateral propodeal area: raised above plical area and indicated by sparser setation. Plical carina: present. Shape of lateral propodeal area: continuous with prespiracular propodeal area. Sculpture of lateral propodeal area: punctate rugulose.

Color of metasoma: yellowish brown to black; reddish brown. Macrosculpture of T1: longitudinally striate; longitudinally strigose. Interstitial sculpture of T1: finely rugulose. Adornment of horn on T1 in female: posteriorly projecting spine. Macrosculpture of T2 in female: longitudinally striate throughout. Macrosculpture of T2 in male: longitudinally striate throughout. Microsculpture on T2: absent. Setal patch of lateral T2: present throughout lateral surface of tergite. Posterior margin of transverse sulcus on T2: straight. Carina along posterior margin of transverse sulcus on T2 in male: present. Carina along posterior margin of transverse sulcus on T2 in female: present. Microsculpture on T3: absent; present. Macrosculpture of T3 medially in female: weakly longitudinally strigose; weakly longitudinally striate. Macrosculpture of T3 laterally in female: longitudinally strigose; longitudinally striate. Macrosculpture of T3 medially in male: longitudinally striate. Macrosculpture of T3 laterally in male: longitudinally striate. Microsculpture on T4: absent. Macrosculpture of T4 medially in female: absent. Macrosculpture of T4 laterally in female: absent. Macrosculpture of T4 in male: longitudinally strigose laterally; absent. Macrosculpture of T5 in female: absent. Constriction of apical T6 in female: present. Punctation of T6 in female: densely and finely punctate throughout. Setation of S1: present as medial tuft. Form of S2 felt field: longitudinal row or patch of setigerous punctures. Macrosculpture of S2 medially: longitudinally striate. Macrosculpture of S3: absent; weakly crenulate to weakly strigose medially.

Wing development: macropterous. Basal vein in hind wing: spectral. Setation of hind wing: reduced anad of submarginal vein. Length of postmarginalis: approximately half of length of stigmalis. RS+M in fore wing: spectral.

#### Diagnosis.

*Paridris aenea* is most similar to *Paridris lemete* (endemic to Puerto Rico). The females of these species are easily separated by the number of basiconic sensilla on A8: one in *Paridris aenea*, and two in *Paridris lemete*; and a carina is present along the crest of the horn of T1 in *Paridris lemete* but not in *Paridris aenea*. The males of *Paridris aenea* may be separated by the coarse rugose or areolate sculpture of the mesopleuron ventral to the femoral depression; in *Paridris lemete* the ventral mesopleuron is mostly smooth with sparse punctation.

#### Link to distribution map.

^18^

#### Associations.

collected near *Prestoea acuminata* var. *montana* (Graham): [Arecales: Arecaceae]

#### Material examined.

*Lectotype* (by present designation), female, *Idris aenea*: **SAINT VINCENT AND THE GRENADINES**: Saint Vincent Island, no date, H. H. Smith, B.M.TYPE HYM. 9.935 (deposited in BMNH). *Paralectotype*, male, *Idris aenea*: **SAINT VINCENT AND THE GRENADINES**: Saint Vincent Island, no date, H. H. Smith, OSUC 397883 (deposited in BMNH). *Lectotype* (by present designation), female, *Caloteleia aenea*: **SAINT VINCENT AND THE GRENADINES**: Saint Vincent Island, no date, H. H. Smith, B.M.TYPE HYM. 9.936 (deposited in BMNH). *Paralectotypes*: 2 males, *Caloteleia aenea*: **SAINT VINCENT AND THE GRENADINES**: OSUC 397892–397893 (deposited in BMNH). *Other material*: (161 females, 144 males) **BELIZE**: 2 females, 5 males, OSUC 181326, 181375–181377, 396509–396510, 396541 (CNCI). **BOLIVIA**: 3 females, 2 males, OSUC 181331, 181396, 181400, 396278–396279 (CNCI). **BRAZIL**: 4 females, 13 males, OSUC 181361, 181364, 181367, 181372, 396066 (CNCI); OSUC 111928, 133058, 133083, 147969, 148060, 225, 254564, 254589, 254592, 266228, 334194, 334198 (OSUC). **COLOMBIA**: 9 females, 21 males, OSUC 181360, 181368, 181402, 396274 (CNCI); OSUC 178136, 181407, 182831, 182833, 185437, 189095, 189243, 192194, 193175, 202090, 256813–256814, 256816, 268895 (IAVH); OSUC 182235, 182835, 188736–188737, 189094, 189244, 191336, 194181, 202091–202092, 256815, 262597 (OSUC). **COSTA RICA**: 36 females, 32 males, OSUC 181302, 181308, 181310, 181317, 181321–181322, 181330, 181335, 181341–181342, 181346–181347, 181350, 181380, 181383, 181387, 181389, 181393, 181405, 334104–334106, 396070–396077, 396085–396086, 396093–396095, 396101, 396104, 396106, 396110–396112, 396115–396116, 396123–396125, 396127–396128, 396275–396277, 396488–396495, 396512, 396540, 396542, 396544, 396547, 396550 (CNCI); OSUC 334190 (OSUC); OSUC 266071, 266073 (TAMU). **CUBA**: 16 females, 4 males, OSUC 334265–334267 (CNCI); OSUC 436213–436227, 436230–436231 (USNM). **DOMINICA**: 2 females, 4 males, OSUC 181338, 396826–396827, 396831–396833 (CNCI). **DOMINICAN REPUBLIC**: 6 females, 3 males, OSUC 181314, 181323, 181325, 181381, 396078, 396088–396089, 396513 (CNCI); OSUC 261872 (OSUC). **ECUADOR**: 10 females, 6 males, OSUC 181315, 181337, 181340, 181345, 181348, 181353–181354, 181369, 181386, 181398, 396092, 396107, 396117–396118, 396120, 396530 (CNCI). **FRENCH GUIANA**: 1 female, 1 male, OSUC 181334, 396545 (CNCI). **GRENADA**: 1 female, OSUC 396830 (CNCI). **GUYANA**: 8 females, 2 males, OSUC 181390, 396263–396267, 396518–396521 (CNCI). **HONDURAS**: 1 female, 2 males, OSUC 334161–334162, 334165 (MZLU). **JAMAICA**: 1 female, OSUC 58703 (OSUC). **MEXICO**: 25 females, 2 males, OSUC 181301, 181303, 181385, 181388, 334073–334085, 334098–334103, 396108–396109, 396511 (CNCI); OSUC 49279 (OSUC). **PANAMA**: 2 females, 13 males, OSUC 181307, 181309, 181318–181320, 181336, 181366, 396079–396081, 396087, 396102–396103, 396105, 396114 (CNCI). **PERU**: 7 females, 15 males, OSUC 181316, 181324, 181343, 181408, 396082–396084, 396119, 396257–396262, 396269, 396280, 396517, 396546 (CNCI); OSUC 237351, 255001–255002 (OSUC); OSUC 232004 (USNM). **SAINT VINCENT AND THE GRENADINES**: 2 females, OSUC 396828–396829 (CNCI). **SURINAME**: 2 females, OSUC 181355–181356 (CNCI). **TRINIDAD AND TOBAGO**: 11 females, 12 males, OSUC 181305, 181362–181363, 396051–396057, 396059–396062, 396067–396068, 396096–396100, 396121–396122 (CNCI).**VENEZUELA**: 12 females, 7 males, OSUC 181306, 181328–181329, 181379, 265174, 334071–334072, 396090–396091, 396113, 396528–396529, 396531–396533, 396543 (CNCI); OSUC 146704, 334201, 79752 (OSUC).

#### Comments.

The large geographical distribution of *Paridris aenea* is accompanied by morphological variation, some of which is correlated with particular regions. Specimens from Cuba and Jamaica have smaller eyes (and consequently a larger OOL) and a pronounced transverse carina on T2 that protrudes laterally, making the anterior width of T2 distinctly greater than the posterior width of T1. Typically, the genal striae do not extend above the midpoint of the eye and are concentrated in the posterior half of the gena. Specimens from Tobago, and some from mainland South America, have elongate genal striae that extend to the vertex, or even around the eye, becoming dorsally continuous with the malar striae. Finally, three female specimens, OSUC 181316, 181345, 334201, have a minute horn on T1. They are otherwise consistent with our concept of *Paridris aenea*, and we consider them to be variants within this species.

**Figures 1–7.**
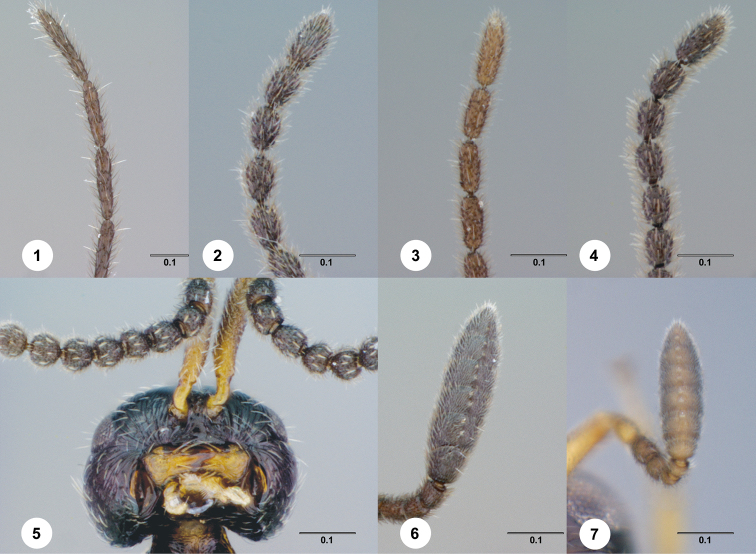
**^79^ 1** *Paridris convexa* sp. n., Antenna, male (OSUC 262116) **2** *Paridris pallipes* (Ashmead), Antenna, male (OSUC 396171) **3** *Paridris dnophos* sp. n., Antenna, male (OSUC 396752) **4** *Paridris aenea* (Ashmead), Antenna, male (OSUC 396540) **5** *Paridris psydrax* sp. n., Head and antennae, ventral view, male (OSUC 181278) **6** *Paridris nayakorum* sp. n., Antennal clava, ventral view, female (OSUC 396697) **7** *Paridris aenea* (Ashmead), Antennal clava, ventral view, female (OSUC 202090)

**Figures 8–13.**
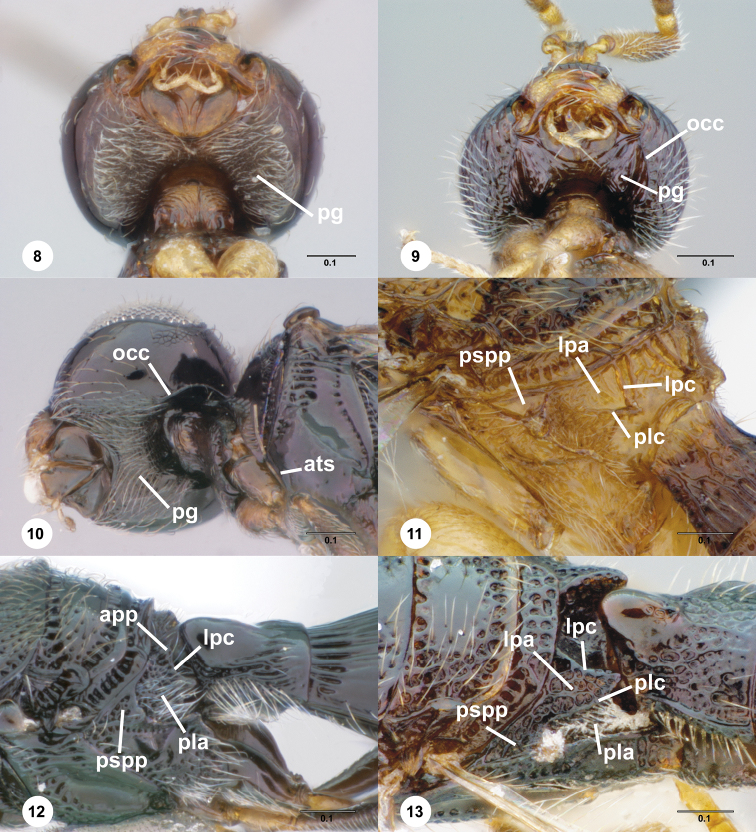
**^80^ 8** *Paridris saurotos* sp. n., head, posterior view, female holotype (OSUC 262111) **9** *Paridris gorn*, head, posterior view, female (OSUC 181261) **10** *Paridris dnophos* sp. n., head, posteroventral view, female (OSUC 190973) **11** *Paridris invicta* sp. n., propodeum, dorsolateral view, male holotype (OSUC 236922) **12** *Paridris dnophos* sp. n., mesoscutellum, metascutellum, propodeum and T1, dorsolateral view, female (OSUC 190971) **13** *Paridris aenea* (Ashmead), mesoscutellum, metascutellum, propodeum and T1, dorsolateral view, female (OSUC 181306)

**Figures 14–17. F3:**
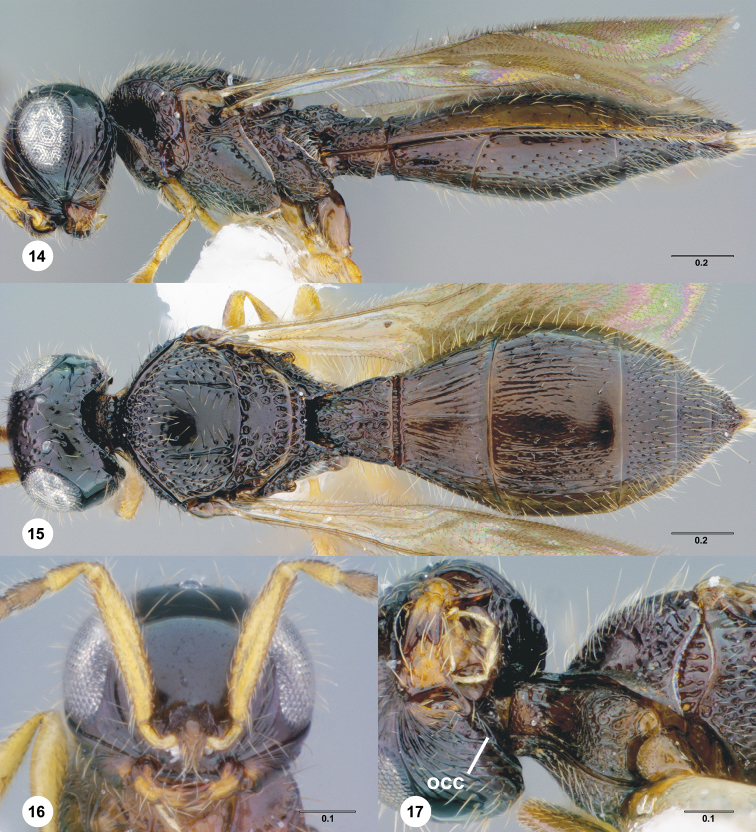
**^81^** *Paridris aenea* (Ashmead) **14** Lateral habitus, female (OSUC 181348) **15** Dorsal habitus, female (OSUC 181348) **16** Head, anterior view, female (OSUC 182833) **17** Head, propleuron and pronotum, ventral view, female (OSUC 396090)

**Figures 18–20.**
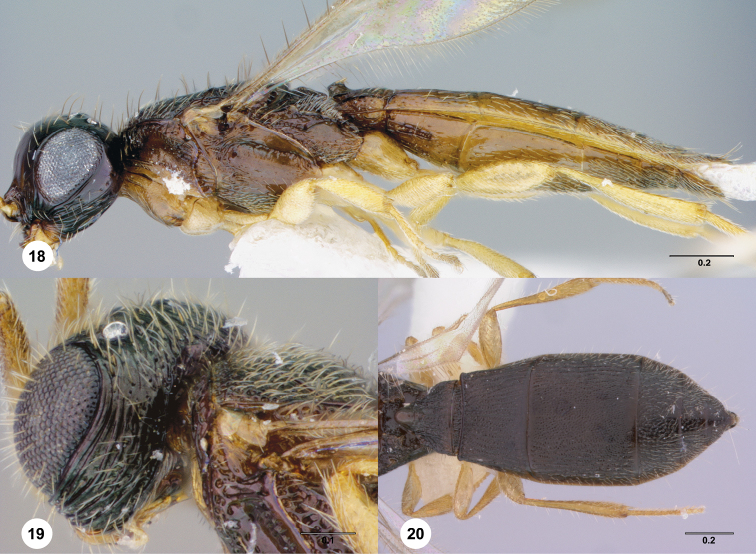
**^82^** *Paridris aenea* (Ashmead) **18** Lateral habitus, female (OSUC 181316) **19** Head, posterolateral view, female (OSUC 181305) **20** Metasoma, dorsal view, female (OSUC 58703).

### Paridris armata

Talamas sp. n.

urn:lsid:zoobank.org:act:A5C4FDC5-ED25-46D6-9AF6-57E3C7F4C289

urn:lsid:biosci.ohio-state.edu:osuc_concepts:298865

http://species-id.net/wiki/Paridris_armata

**Figures 21–25.**
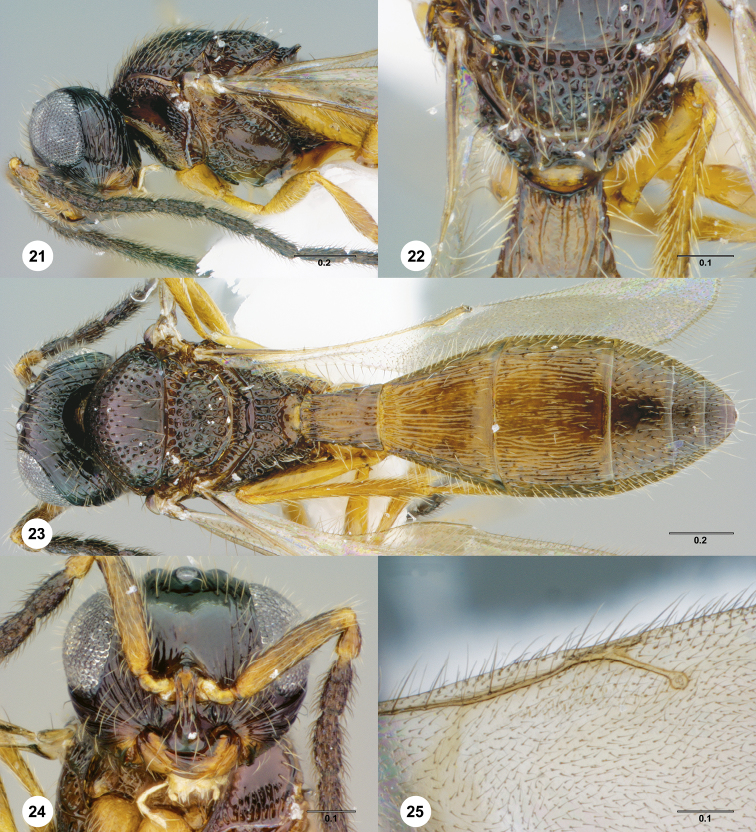
**^83^** *Paridris armata*, sp. n., male holotype (OSUC 181352) **21** Head and mesosoma, lateral view **22** Mesoscutellum, metascutellum and T1, dorsal view **23** Dorsal habitus **24** Head, anterior view **25** Venation of forewing, dorsal view

Figures 21–25 Morphbank^19^

#### Description. 

Male body length: 2.35 mm (n=1).

Color of head: black. Distal margin of clypeus: smooth. Width of clypeus: equal to or less than width of interantennal process. Lateral corner of clypeus: rounded. Development of interantennal process ventrally: connecting with clypeus. Number of mandibular teeth: three. Length of mediofacial striae: extending to dorsal frons. Shape of gena in dorsal view: moderately receding behind compound eye. Striae on gena: pronounced. Length of striae on gena: extending above ventral margin of eye. Distribution of microsculpture on head: absent. Length of OOL: less than 2 ocellar diameters. Occipital carina above foramen magnum: present. Anterior margin of occipital carina: comprised of medium to large sized cells. Setation of postgena: sparse. Ventral extent of occipital carina: extending to base of mandible.

Color of mesosoma: reddish brown. Dorsal half of pronotal cervical sulcus: present as line of small to minute cells. Ventral half of pronotal cervical sulcus: present as line of small to minute cells. Transverse pronotal carina: present in posterodorsal corner of pronotum. Shape of pronotal shoulder in dorsal view: without dorsal surface. Form of pronotal suprahumeral sulcus: punctate rugulose. Macrosculpture of anterior medial mesoscutum: absent. Density of punctation on anterior medial mesoscutum: dense throughout. Reticulate microfissures on anterior half of medial mesoscutum: absent. Pustulate microsculpture on anterior mesoscutum: absent. Density of punctation on posterior medial mesoscutum: sparse. Notaulus: present as cluster of punctures at posterior margin of mesoscutum. Macrosculpture of mesoscutellum: punctate rugose. Postacetabular sulcus: crenulate. Mesopleural carina: absent. Punctures on posterodorsal mesepimeral area: absent. Sculpture of mesopleuron anteroventral to femoral depression: densely punctate anteriorly, smooth posteriorly and on ventral surface. Sculpture of posterior mesepimeral area: smooth. Form of metascutellum in male: bispinose. Paracoxal and metapleural sulci: uncertain, separate. Posterior margin of metapleuron below propodeal spiracle: straight to moderately convex. Setation between metapleural triangle and metapleural sulcus: absent. Sculpture between metapleural triangle and metapleural sulcus: punctate rugose. Sculpture of metapleural triangle: punctate rugose. Setation of metapleural triangle: sparse. Anterior propodeal projection: absent. Setation of metasomal depression: absent. Lateral propodeal area: indicated by sparser degree of setation. Plical carina: absent. Shape of lateral propodeal area: continuous with prespiracular propodeal area. Sculpture of lateral propodeal area: rugose.

Color of metasoma: brown. Macrosculpture of T1: longitudinally strigose. Interstitial sculpture of T1: smooth. Macrosculpture of T2 in male: weakly longitudinally striate throughout. Microsculpture on T2: absent. Setal patch of lateral T2: present throughout lateral surface of tergite. Posterior margin of transverse sulcus on T2: weakly convex. Carina along posterior margin of transverse sulcus on T2 in male: absent. Microsculpture on T3: absent. Macrosculpture of T3 medially in male: weakly longitudinally striate. Macrosculpture of T3 laterally in male: weakly longitudinally striate. Microsculpture on T4: absent. Macrosculpture of T4 in male: absent. Setation of S1: absent. Form of S2 felt field: longitudinal row or patch of setigerous punctures. Macrosculpture of S2 medially: absent. Macrosculpture of S3: absent.

Wing development: macropterous. Basal vein in hind wing: spectral. Setation of hind wing: uniform throughout. Length of postmarginalis: less than half of length of stigmalis. RS+M in fore wing: nebulous.

#### Diagnosis.

*Paridris armata* is not acutely similar to any of the other *Paridris* species in the New World. The bispinose shape of the metascutellum and very narrow clypeus unambiguously separate it from the other species treated here.

#### Etymology.

The adjectival Latin epithet “armata” is given to this species for the shape and relatively large size of the metascutellum.

#### Link to distribution map.

**^20^**

#### Material examined.

*Holotype*, male: **VENEZUELA**: Bolívar St., camp, Auyán Tepuy, 05°46'07"N, 62°31'56"W, 2075m, 19.IV–25.IV.1994, yellow pan trap, L. Masner & J. L. Garcia, OSUC 181352 (deposited in CNCI).

### Paridris convexa

Talamas sp. n.

urn:lsid:zoobank.org:act: 35B139C7-8B3E-44A6-80D7-08141057B48F

urn:lsid:biosci.ohio-state.edu:osuc_concepts:299093

http://species-id.net/wiki/Paridris_convexa

Figures 1 26–29 82 Morphbank^21^

#### Description.

Female body length: 1.19–1.51 mm (n=4). Male body length: 1.26–2.44 mm (n=12).

Number of basiconic sensilla on A8: 1.

Color of head: brown; black. Distal margin of clypeus: smooth. Width of clypeus: wider than interantennal process. Lateral corner of clypeus: projecting into acute angle. Development of interantennal process ventrally: not reaching clypeus. Number of mandibular teeth: three. Length of mediofacial striae: not extending above midpoint of eye. Shape of gena in dorsal view: moderately receding behind compound eye. Striae on gena: weakly indicated. Length of striae on gena: terminating below ventral margin of eye. Form of microsculpture on head: reticulate microfissures. Distribution of microsculpture on head: present throughout dorsal head. Length of OOL: greater than 2 ocellar diameters. Occipital carina above foramen magnum: present. Anterior margin of occipital carina: simple. Setation of postgena: dense. Ventral extent of occipital carina: absent below midpoint of foramen magnum.

Color of mesosoma: reddish brown; yellowish brown. Dorsal half of pronotal cervical sulcus: present as line of small to minute cells. Ventral half of pronotal cervical sulcus: present as line of small to minute cells. Transverse pronotal carina: absent. Shape of pronotal shoulder in dorsal view: without dorsal surface. Form of pronotal suprahumeral sulcus: sparsely punctate; line of uniform punctures. Macrosculpture of anterior medial mesoscutum: absent. Density of punctation on anterior medial mesoscutum: sparse; moderate. Reticulate microfissures on anterior half of medial mesoscutum: present throughout. Density of punctation on posterior medial mesoscutum: sparse; absent. Notaulus: percurrent, reaching suprahumeral sulcus as a smooth furrow; abbreviate, not reaching mesoscutal suprahumeral sulcus. Orientation of notauli: parallel. Shape of notaulus at posterior apex: parallel-sided. Macrosculpture of mesoscutellum: absent. Postacetabular sulcus: crenulate. Mesopleural carina: absent. Punctures on posterodorsal mesepimeral area: absent. Sculpture of mesopleuron anteroventral to femoral depression: densely punctate; areolate to punctate rugose throughout. Sculpture of posterior mesepimeral area: smooth. Form of metascutellum in female: transverse lamella, posterior margin convex. Form of metascutellum in male: transverse lamella, pointed medially; transverse lamella, posterior margin convex. Paracoxal and metapleural sulci: fused. Setation between metapleural triangle and metapleural sulcus: present throughout. Sculpture of metapleural triangle: punctate rugose. Setation of metapleural triangle: sparse. Anterior propodeal projection: absent. Setation of metasomal depression: present. Lateral propodeal area: undifferentiated from plical area. Plical carina: absent.

Color of metasoma: reddish brown; yellowish brown. Macrosculpture of T1: longitudinally striate. Interstitial sculpture of T1: finely rugulose. Adornment of horn on T1 in female: transverse ridge at base of horn. Macrosculpture of T2 in female: longitudinally striate throughout. Macrosculpture of T2 in male: longitudinally striate throughout. Microsculpture on T2: absent. Setal patch of lateral T2: present throughout lateral surface of tergite. Posterior margin of transverse sulcus on T2: distinctly convex. Carina along posterior margin of transverse sulcus on T2 in male: absent. Carina along posterior margin of transverse sulcus on T2 in female: absent. Microsculpture on T3: absent. Macrosculpture of T3 medially in female: absent. Macrosculpture of T3 laterally in female: longitudinally striate. Macrosculpture of T3 medially in male: absent. Macrosculpture of T3 laterally in male: longitudinally striate. Microsculpture on T4: absent. Macrosculpture of T4 medially in female: absent. Macrosculpture of T4 laterally in female: absent. Macrosculpture of T4 in male: absent. Macrosculpture of T5 in female: absent. Constriction of apical T6 in female: present. Setation of S1: densely present throughout. Form of S2 felt field: line of dense setae along longitudinal ridge. Macrosculpture of S2 medially: longitudinally striate.

Wing development: macropterous. Basal vein in hind wing: spectral. Setation of hind wing: uniform throughout. RS+M in fore wing: nebulous.

#### Diagnosis.

*Paridris convexa* is most similar to *Paridris saurotos* and *Paridris isabelicae*. Males and females of *Paridris convexa* may be separated from these two species by the presence of reticulate microfissures through the head. In *Paridris saurotos* and *Paridris isabelicae* this microsculpture is limited to patches on the temple, between the median and lateral ocelli, and directly posterior to the lateral ocellus.

#### Etymology.

The Latin epithet “convexa” is adjectival, meaning rounded or smooth. It is given to this species for its smooth surface sculpture.

#### Link to distribution map.

**^22^**

#### Material examined.

*Holotype*, female: **COSTA RICA**: Heredia Prov., La Selva Biological Station, 10°26'N, 84°01'W, 75m, 27.II–28.II.2003, sweeping, J. S. Noyes, OSUC 181392 (deposited in BMNH). *Paratypes*: (3 females, 13 males) **COSTA RICA**: 3 females, 9 males, OSUC 181304, 181327, 181382, 181403–181404, 181409, 262112–262114, 265168 (CNCI); OSUC 181391, 181399 (OSUC). **PANAMA**: 4 males, OSUC 262115–262116 (CNCI); OSUC 181332, 262117 (OSUC).

**Figures 26–29.**
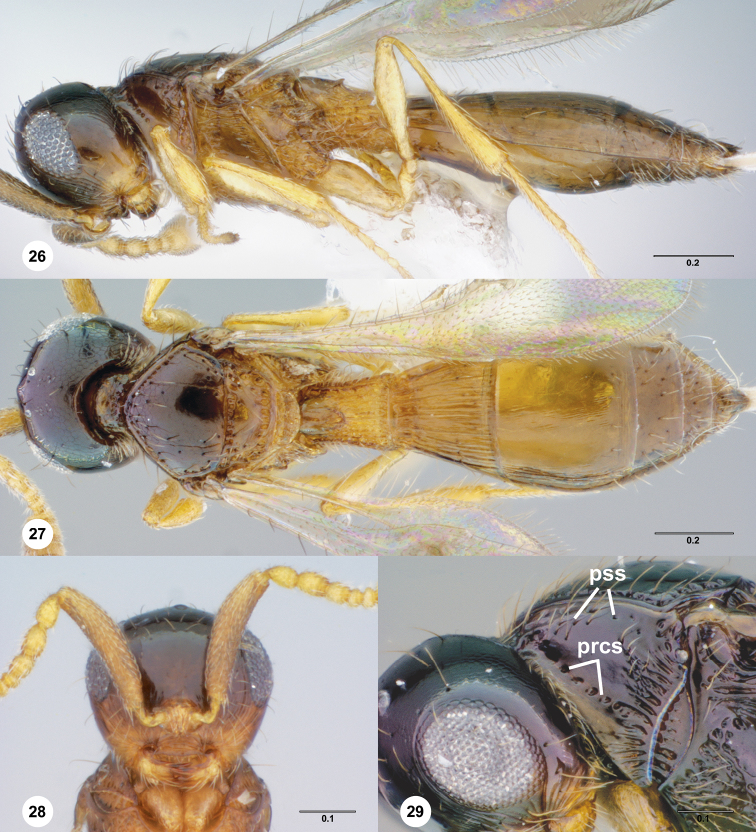
**^84^** *Paridris convexa*, sp. n. **26** Lateral habitus, female holotype (OSUC 181392) **27** Dorsal habitus, female holotype (OSUC 181392) **28** Head, anterior view, female (OSUC 262112) **29** Head and pronotum, lateral view, male (OSUC 262115)

### Paridris dnophos

Talamas sp. n.

urn:lsid:zoobank.org:act: 79588AE4-3C13-49DE-B0AD-ADDC7960179B

urn:lsid:biosci.ohio-state.edu:osuc_concepts:299226

http://species-id.net/wiki/Paridris_dnophos

Figures 3 10, 12 30–33 Morphbank^23^

#### Description.

Female body length: 1.21–1.97 mm (n=21). Male body length: 1.08–1.88 mm (n=20).

Number of basiconic sensilla on A8: 1.

Color of head: brown; black. Distal margin of clypeus: serrate. Width of clypeus: wider than interantennal process. Lateral corner of clypeus: projecting into acute angle. Development of interantennal process ventrally: not reaching clypeus. Number of mandibular teeth: three. Length of mediofacial striae: not extending above midpoint of eye. Shape of gena in dorsal view: strongly receding behind compound eye. Striae on gena: weakly indicated. Length of striae on gena: terminating below midpoint of eye. Form of microsculpture on head: reticulate microfissures. Distribution of microsculpture on head: present only on anterodorsal margin of eye, temples, and posterior to lateral ocellus. Length of OOL: less than 2 ocellar diameters. Occipital carina above foramen magnum: present. Anterior margin of occipital carina: comprised of small to miniscule cells; simple; faintly crenulate throughout. Setation of postgena: dense. Ventral extent of occipital carina: absent below midpoint of foramen magnum.

Color of mesosoma: brown; black. Dorsal half of pronotal cervical sulcus: present as line of small to minute cells. Ventral half of pronotal cervical sulcus: present as line of small to minute cells; present as line of large cells. Transverse pronotal carina: present in posterodorsal corner of pronotum. Shape of pronotal shoulder in dorsal view: without dorsal surface. Form of pronotal suprahumeral sulcus: broadly punctate; punctate rugulose; line of uniform punctures. Macrosculpture of anterior medial mesoscutum: absent. Density of punctation on anterior medial mesoscutum: dense along mesoscutal suprahumeral sulcus, otherwise sparse; sparse; moderate; dense throughout. Reticulate microfissures on anterior half of medial mesoscutum: absent. Pustulate microsculpture on anterior mesoscutum: absent. Density of punctation on posterior medial mesoscutum: sparse; dense; moderately dense. Notaulus: percurrent, reaching suprahumeral sulcus as a line of punctures; abbreviate, not reaching mesoscutal suprahumeral sulcus. Orientation of notauli: parallel. Shape of notaulus at posterior apex: parallel-sided. Macrosculpture of mesoscutellum: absent. Postacetabular sulcus: comprised of distinct, closed cells. Mesopleural carina: absent. Punctures on posterodorsal mesepimeral area: absent. Sculpture of mesopleuron anteroventral to femoral depression: densely punctate; smooth; moderately punctate; densely punctate on lateral surface, smooth on ventral surface. Sculpture of posterior mesepimeral area: smooth. Form of metascutellum in female: transverse lamella, pointed medially; obscured by horn of T1. Form of metascutellum in male: transverse lamella, pointed medially. Paracoxal and metapleural sulci: fused. Posterior margin of metapleuron below propodeal spiracle: with blunt angle near intersection with metapleural sulcus. Setation between metapleural triangle and metapleural sulcus: present throughout. Sculpture between metapleural triangle and metapleural sulcus: punctate. Sculpture of metapleural triangle: densely punctate. Setation of metapleural triangle: dense. Anterior propodeal projection: present. Setation of metasomal depression: present. Lateral propodeal area: undifferentiated from plical area. Plical carina: absent.

Color of metasoma: brown; black; yellow anteriorly, brown posteriorly. Macrosculpture of T1: longitudinally striate. Interstitial sculpture of T1: smooth. Adornment of horn on T1 in female: absent. Macrosculpture of T2 in female: longitudinally and sparsely striate, medial striae not reaching posterior margin. Macrosculpture of T2 in male: longitudinally striate anteriorly, smooth posteriorly. Microsculpture on T2: absent. Setal patch of lateral T2: present in thin line along lateral edge. Posterior margin of transverse sulcus on T2: distinctly convex. Carina along posterior margin of transverse sulcus on T2 in male: absent. Carina along posterior margin of transverse sulcus on T2 in female: absent. Microsculpture on T3: absent. Macrosculpture of T3 medially in female: absent; finely and densely punctate. Macrosculpture of T3 laterally in female: absent. Macrosculpture of T3 medially in male: absent. Macrosculpture of T3 laterally in male: absent. Microsculpture on T4: absent. Macrosculpture of T4 medially in female: absent. Macrosculpture of T4 laterally in female: absent. Macrosculpture of T4 in male: absent. Macrosculpture of T5 in female: absent. Constriction of apical T6 in female: absent. Punctation of T6 in female: sparse along longitudinal midline and anterior margin, dense and fine laterally. Setation of S1: densely present throughout. Form of S2 felt field: line of dense setae along longitudinal ridge. Macrosculpture of S2 medially: longitudinally striate. Macrosculpture of S3: absent.

Wing development: macropterous. Basal vein in hind wing: nebulous. Setation of hind wing: uniform throughout. Length of postmarginalis: punctiform. RS+M in fore wing: nebulous.

#### Diagnosis.

*Paridris dnophos* may be strikingly similar to *Paridris nayakorum* in coloration and shape of the body. Females may easily be separated by having only 1 basiconic sensillum on A8, versus 2 in *Paridris nayakorum*; by the absence of striation on T1; and the linear form of the posterior notaulus. The presence of an externally visible metascutellum serves well to separate *Paridris dnophos* in most cases, but this character should not be used alone given that a few specimens of have a large horn on T1 and reduced metascutellum. **Etymology**. The Greek epithet “dnophos” means “darkness” and is given to this species for the color of its body. The name is treated as a noun in apposition.

#### Link to distribution map.

^24^

#### Material examined.

*Holotype*, female: **COLOMBIA**: Magdalena Dept., Nevada de Santa Marta Mts., M.602, El Ramo, 10°48'N, 73°39'W, 2500m, 16.VIII–31.VIII.2000, Malaise trap, J. Cantillo, OSUC 191490 (deposited in IAVH). *Paratypes*: (378 females, 171 males) **BELIZE**: 1 male, OSUC 396702 (CNCI). **BOLIVIA**: 6 females, 1 male, OSUC 396500–396506 (CNCI).**BRAZIL**: 11 females, 6 males, OSUC 396460–396466 (CNCI); OSUC 10784, 134086, 134501, 134639, 134819, 135102, 135623, 135736, 135771, 13589 (OSUC). **COLOMBIA**: 75 females, 63 males, OSUC 396298–396348, 396600–396603, 396647–396650, 396683–396686, 396711–396712, 396731, 396750–396751, 396771–396772, 405110 (CNCI); OSUC 144070–144071, 182832, 190858–190859, 190972–190973, 190976–190977, 191484–191485, 191488–191489, 191492, 256817–256818, 262129, 265241–265242, 266115–266121, 266124–266125, 268899, 334195, 334200, 396594–396599, 396604–396605, 396729 (IAVH); OSUC 144067–144069, 144124, 190971, 190974–190975, 190978, 191486–191487, 191491, 192195, 193122–193123, 193236, 265243, 266114, 266122–266123, 268896–268898, 269528, 334196–334197, 334199, 372630 (OSUC). **COSTA RICA**: 94 females, 29 males, OSUC 262123–262124, 262127, 396350–396351, 396354–396355, 396359–396361, 396363, 396374–396375, 396377, 396379, 396382–396386, 396411–396416, 396422, 396429, 396431, 396434, 396450–396459, 396467–396487, 396507–396508, 396526–396527, 396534, 396549, 396551–396552, 396555, 396557–396560, 396678–396679, 396682, 396695, 396698, 396704–396706, 396709–396710, 396720, 396725–396727, 396732–396737, 396745, 396752, 396754, 396757–396769, 396773–396782, 396800, 396812 (CNCI); OSUC 334191 (INBC). **ECUADOR**: 24 females, 50 males, OSUC 262125–262126, 262133, 262141–262144, 396364–396370, 396421, 396433, 396514–396516, 396536–396537, 396614–396625, 396646, 396651–396672, 396674–396676, 396770, 396783–396789, 396794–396795, 396801, 396807, 396811, 396813–396814 (CNCI). **EL SALVADOR**: 40 females, OSUC 396398–396405, 396423, 396432, 396538, 396582–396593, 396626–396637, 396713–396714, 396721–396722, 396809 (CNCI).**FRENCH GUIANA**: 3 females, 2 males, OSUC 396715–396719 (CNCI). **GUATEMALA**: 1 female, OSUC 396753 (CNCI). **HONDURAS**: 4 females, 3 males, OSUC 396742–396743 (CNCI); OSUC 334159–334160, 334163–334164, 334166 (MZLU). **MEXICO**: 2 females, 2 males, OSUC 396792–396793, 396803–396804 (CNCI). **NICARAGUA**: 9 females, 6 males, OSUC 396567–396581 (CNCI). **PANAMA**: 37 females, 4 males, OSUC 160254 (AMNH); OSUC 262137–262139, 396380–396381, 396387–396397, 396418–396420, 396425–396428, 396430, 396523–396525, 396535, 396539, 396687–396690, 396699–396701, 396723–396724, 396808 (CNCI); OSUC 334143 (TAMU). **PERU**: 3 females, OSUC 396728, 396790–396791 (CNCI). **TRINIDAD AND TOBAGO**: 17 females, 3 males, OSUC 262132, 396376, 396606–396613, 396703, 396738–396741, 396744, 396746–396747, 396806, 396815 (CNCI). **VENEZUELA**: 52 females, 1 male, OSUC 262130–262131, 262135, 262140, 396371–396373, 396378, 396406–396410, 396417, 396424, 396435–396436, 396561–396566, 396638–396645, 396691–396694, 396696, 396707–396708, 396730, 396748–396749, 396755–396756, 396796–396799, 396802, 396805, 396816–396817 (CNCI); OSUC 334192–334193 (OSUC).

#### Comments.

Morphological variation within *Paridris dnophos* occurs primarily in color and density of setation and punctation on the head and mesosoma. The antennae, legs, T1, and anterior T2 range from black to yellow; the head, mesosoma and remainder of the metasoma vary from black to brown. The setation of the head and dorsal mesosoma varies from white to golden yellow and may be extremely sparse to dense. The density of punctation and setation of the lateral pronotum are similarly variable.

**Figures 30–33.**
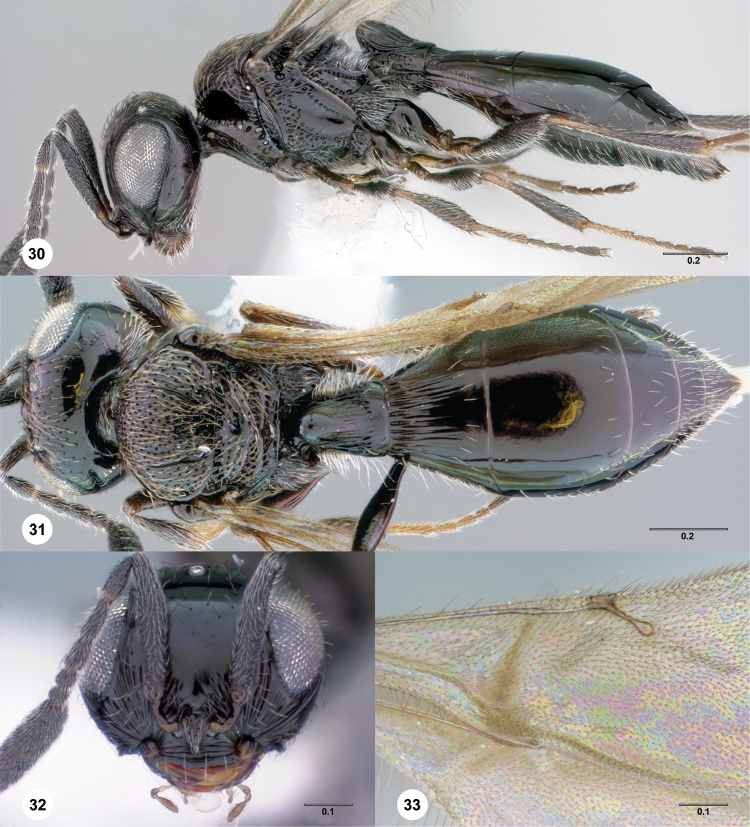
**^85^** *Paridris dnophos*, sp. n. **30** Lateral habitus, female holotype (OSUC 191490) **31** Dorsal habitus, female (OSUC 191488) **32** Head, anterior view, female (OSUC 190977) **33** Venation of forewing, dorsal view, female holotype (OSUC 191490)

### Paridris gongylos

Talamas & Masner sp. n.

urn:lsid:zoobank.org:act: 4B4388FC-3D6D-469E-82A1-A4CCB6507E12

urn:lsid:biosci.ohio-state.edu:osuc_concepts:284313

http://species-id.net/wiki/Paridris_gongylos

**Figures 34–37.**
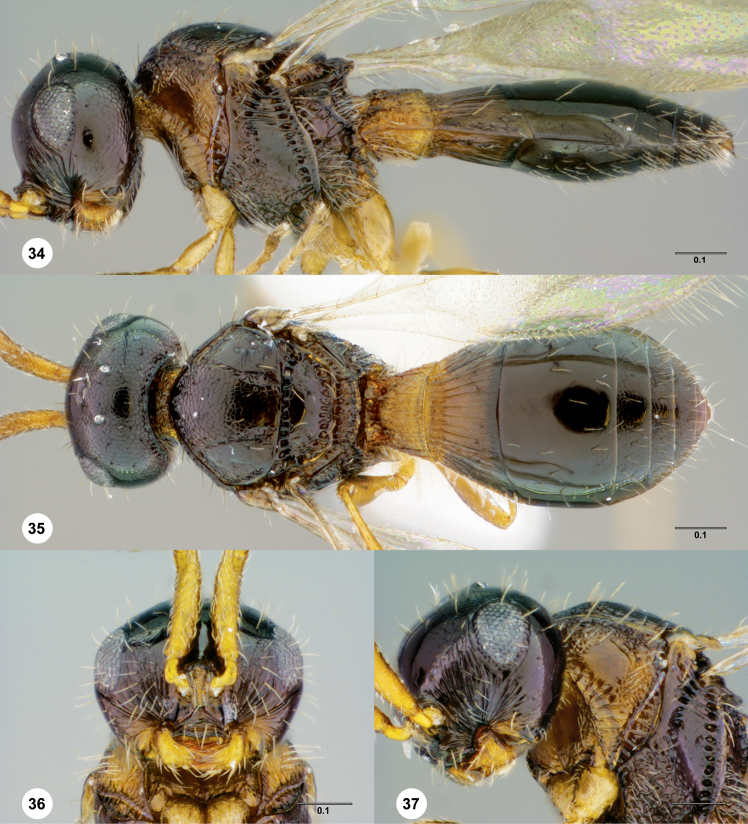
**^86^** *Paridris gongylos*, sp. n. **34** Lateral habitus, male holotype (OSUC 334015) **35** Dorsal habitus, male holotype (OSUC 334015) **36** Head, anterior view, male (OSUC 334016) **37** Head, propleuron and pronotum, anterolateral view (OSUC 334016).

Figures 34–37 Morphbank^25^

#### Description.

Male body length: 1.17–1.47 mm (n=20).

Color of head: black. Distal margin of clypeus: serrate. Width of clypeus: wider than interantennal process. Lateral corner of clypeus: projecting into acute angle. Development of interantennal process ventrally: not reaching clypeus. Number of mandibular teeth: three. Length of mediofacial striae: not extending above midpoint of eye. Shape of gena in dorsal view: not receding or slightly bulging directly behind compound eye. Striae on gena: weakly indicated. Length of striae on gena: terminating below ventral margin of eye. Form of microsculpture on head: reticulate microfissures. Distribution of microsculpture on head: present throughout dorsal head. Length of OOL: greater than 2 ocellar diameters. Occipital carina above foramen magnum: present. Anterior margin of occipital carina: crenulate. Setation of postgena: dense. Ventral extent of occipital carina: absent below midpoint of foramen magnum.

Color of mesosoma: reddish brown. Dorsal half of pronotal cervical sulcus: present as line of small to minute cells. Ventral half of pronotal cervical sulcus: present as line of large cells. Transverse pronotal carina: absent. Shape of pronotal shoulder in dorsal view: narrow and striplike. Form of pronotal suprahumeral sulcus: punctate rugulose. Macrosculpture of anterior medial mesoscutum: absent. Density of punctation on anterior medial mesoscutum: sparse. Reticulate microfissures on anterior half of medial mesoscutum: present throughout. Pustulate microsculpture on anterior mesoscutum: absent. Density of punctation on posterior medial mesoscutum: absent. Notaulus: percurrent, reaching suprahumeral sulcus as a smooth furrow; abbreviate, not reaching mesoscutal suprahumeral sulcus. Orientation of notauli: converging posteriorly. Shape of notaulus at posterior apex: ovoid. Macrosculpture of mesoscutellum: absent; rugulose laterally, smooth medially. Postacetabular sulcus: crenulate. Mesopleural carina: absent. Punctures on posterodorsal mesepimeral area: absent. Sculpture of mesopleuron anteroventral to femoral depression: areolate to punctate rugose throughout. Sculpture of posterior mesepimeral area: rugulose. Form of metascutellum in male: transverse lamella, posterior margin convex. Paracoxal and metapleural sulci: fused. Posterior margin of metapleuron below propodeal spiracle: with blunt angle near intersection with metapleural sulcus. Setation between metapleural triangle and metapleural sulcus: absent. Sculpture between metapleural triangle and metapleural sulcus: punctate rugose. Sculpture of metapleural triangle: punctate rugose. Setation of metapleural triangle: dense. Anterior propodeal projection: absent. Setation of metasomal depression: absent. Lateral propodeal area: undifferentiated from plical area. Plical carina: absent.

Color of metasoma: yellow anteriorly, brown posteriorly. Macrosculpture of T1: longitudinally strigose. Interstitial sculpture of T1: finely rugulose. Macrosculpture of T2 in male: longitudinally striate throughout. Microsculpture on T2: absent. Setal patch of lateral T2: present in thin line along lateral edge. Posterior margin of transverse sulcus on T2: weakly convex. Carina along posterior margin of transverse sulcus on T2 in male: absent. Microsculpture on T3: absent. Macrosculpture of T3 medially in male: absent. Macrosculpture of T3 laterally in male: absent. Microsculpture on T4: absent. Macrosculpture of T4 in male: absent. Setation of S1: present throughout, moderately dense. Form of S2 felt field: line of dense setae along longitudinal ridge. Macrosculpture of S2 medially: longitudinally striate. Macrosculpture of S3: absent.

Wing development: macropterous. Basal vein in hind wing: spectral. Setation of hind wing: reduced anad of submarginal vein. Length of postmarginalis: punctiform. RS+M in fore wing: spectral.

#### Diagnosis.

*Paridris gongylos* is closest morphologically with *Paridris pallipes* with which it shares the presence of very fine reticulate fissures throughout the dorsal head and mesosoma. The males, from which this species is known, have a crenulate occipital rim that distinguishes them from males of *Paridris pallipes*.

#### Etymology.

The Greek word gongylos, meaning “rounded”, is given to this species for the shape of its head and the curves of its metasoma. The epithet is treated as a noun in apposition.

#### Link to distribution map.

**^26^**

#### Material examined.

*Holotype*, male: **UNITED STATES**: TN, Blount Co., Top of the World, old growth pine, Great Smoky Mountains National Park, 35°38'N, 83°55'W, 670m, 30.VII–13.VIII.1998, Malaise trap, H. Alley, OSUC 334015 (deposited in CNCI). *Paratypes*: **UNITED STATES**: 22 males, OSUC 181281–181282, 334016, 334018, 334020–334028, 334268–334272 (CNCI); OSUC 181280, 334017, 334019, 334273 (OSUC).

### Paridris gorn

Talamas & Masner sp. n.

urn:lsid:zoobank.org:act: 3AFAB6DC-FF98-4CF4-A0C5-2AD9085C13D1

urn:lsid:biosci.ohio-state.edu:osuc_concepts:299227

http://species-id.net/wiki/Paridris_gorn

Figures 9 38–41 79 Morphbank^27^

#### Description.

Female body length: 1.74–2.00 mm (n=20). Male body length: 1.75 mm (n=1).

Number of basiconic sensilla on A8: 1.

Color of head: brown; black. Distal margin of clypeus: serrate. Width of clypeus: wider than interantennal process. Lateral corner of clypeus: projecting into acute angle. Development of interantennal process ventrally: not reaching clypeus. Number of mandibular teeth: three. Length of mediofacial striae: not extending above midpoint of eye; extending to dorsal frons. Shape of gena in dorsal view: moderately receding behind compound eye. Striae on gena: pronounced. Length of striae on gena: extending above ventral margin of eye. Distribution of microsculpture on head: absent. Length of OOL: less than 2 ocellar diameters. Occipital carina above foramen magnum: present. Anterior margin of occipital carina: comprised of medium to large sized cells. Setation of postgena: sparse. Ventral extent of occipital carina: extending to base of mandible.

Color of mesosoma: brown; black. Dorsal half of pronotal cervical sulcus: present as smooth furrow. Ventral half of pronotal cervical sulcus: present as line of small to minute cells; present as line of large cells. Transverse pronotal carina: present in posterior half of pronotum. Shape of pronotal shoulder in dorsal view: narrow and striplike. Form of pronotal suprahumeral sulcus: areolate. Macrosculpture of anterior medial mesoscutum: absent; irregularly rugulose. Density of punctation on anterior medial mesoscutum: dense along mesoscutal suprahumeral sulcus, otherwise sparse. Reticulate microfissures on anterior half of medial mesoscutum: absent. Pustulate microsculpture on anterior mesoscutum: present. Density of punctation on posterior medial mesoscutum: moderately dense. Notaulus: percurrent, reaching suprahumeral sulcus as a smooth furrow; percurrent, reaching suprahumeral sulcus as a line of punctures. Orientation of notauli: converging posteriorly. Shape of notaulus at posterior apex: ovoid. Macrosculpture of mesoscutellum: punctate rugose along margins, smooth medially. Postacetabular sulcus: crenulate. Mesopleural carina: absent; present only anterodorsally. Punctures on posterodorsal mesepimeral area: large. Sculpture of mesopleuron anteroventral to femoral depression: areolate to punctate rugose throughout. Sculpture of posterior mesepimeral area: smooth. Form of metascutellum in female: bispinose. Form of metascutellum in male: bispinose. Paracoxal and metapleural sulci: separate. Setation between metapleural triangle and metapleural sulcus: absent. Sculpture between metapleural triangle and metapleural sulcus: punctate rugose. Sculpture of metapleural triangle: punctate rugose. Setation of metapleural triangle: moderately dense. Anterior propodeal projection: absent. Setation of metasomal depression: absent. Lateral propodeal area: indicated by sparser degree of setation. Plical carina: indistinguishable from propodeal sculpture except at posterior apex; present. Shape of lateral propodeal area: continuous with prespiracular propodeal area. Sculpture of lateral propodeal area: punctate rugulose.

Color of metasoma: brown; black. Macrosculpture of T1: longitudinally striate. Interstitial sculpture of T1: finely rugulose. Adornment of horn on T1 in female: absent. Macrosculpture of T2 in female: longitudinally striate throughout. Macrosculpture of T2 in male: longitudinally striate throughout. Microsculpture on T2: absent. Setal patch of lateral T2: present throughout lateral surface of tergite. Posterior margin of transverse sulcus on T2: straight. Carina along posterior margin of transverse sulcus on T2 in male: present. Carina along posterior margin of transverse sulcus on T2 in female: present. Microsculpture on T3: present. Macrosculpture of T3 medially in female: weakly longitudinally strigose; absent. Macrosculpture of T3 laterally in female: longitudinally strigose. Macrosculpture of T3 medially in male: absent; weakly longitudinally striate. Macrosculpture of T3 laterally in male: longitudinally striate. Microsculpture on T4: absent. Macrosculpture of T4 medially in female: absent. Macrosculpture of T4 laterally in female: absent. Macrosculpture of T4 in male: absent. Macrosculpture of T5 in female: absent. Constriction of apical T6 in female: present. Punctation of T6 in female: densely and finely punctate throughout. Setation of S1: absent. Form of S2 felt field: longitudinal row or patch of setigerous punctures. Macrosculpture of S2 medially: longitudinally striate. Macrosculpture of S3: absent.

Wing development: macropterous. Basal vein in hind wing: spectral. Setation of hind wing: reduced anad of submarginal vein. Length of postmarginalis: approximately equal to length of stigmalis. RS+M in fore wing: nebulous.

#### Diagnosis.

*Paridris gorn* is most similar *to P. soucouyant*, particularly in the bispinose shape of the metascutellum and punctate-rugose sculpture of the head. These two species may be separated by the sculpture of T4–T5: punctate-rugose in *Paridris soucouyant*, smooth in *Paridris gorn*. Additionally, females of *Paridris gorn* have a horn on T1 that is either smooth or has shallow punctures along its longitudinal midline. In *Paridris soucouyant* a carina is present along the dorsal crest of the horn.

#### Etymology.

This species is named after a reptilian alien race from the original Star Trek television series for the similar appearance of their compound eyes. The epithet is treated as a noun in apposition.

#### Link to distribution map.

**^28^**

#### Material examined.

*Holotype*, female: **UNITED STATES**: OH, Franklin Co., vegetation / along railroad tracks, Columbus, 39°59'21"N, 82°59'41"W, 11.VI–13.VI.2011, yellow pan trap, E. Talamas, OSUC 405092 (deposited in OSUC). *Paratypes*: **UNITED STATES**: 45 females, 2 males, OSUC 181260–181267, 334048–334070, 396553–396554 (CNCI); OSUC 256459, 405080–405091, 405109 (OSUC).

**Figures 38–41.**
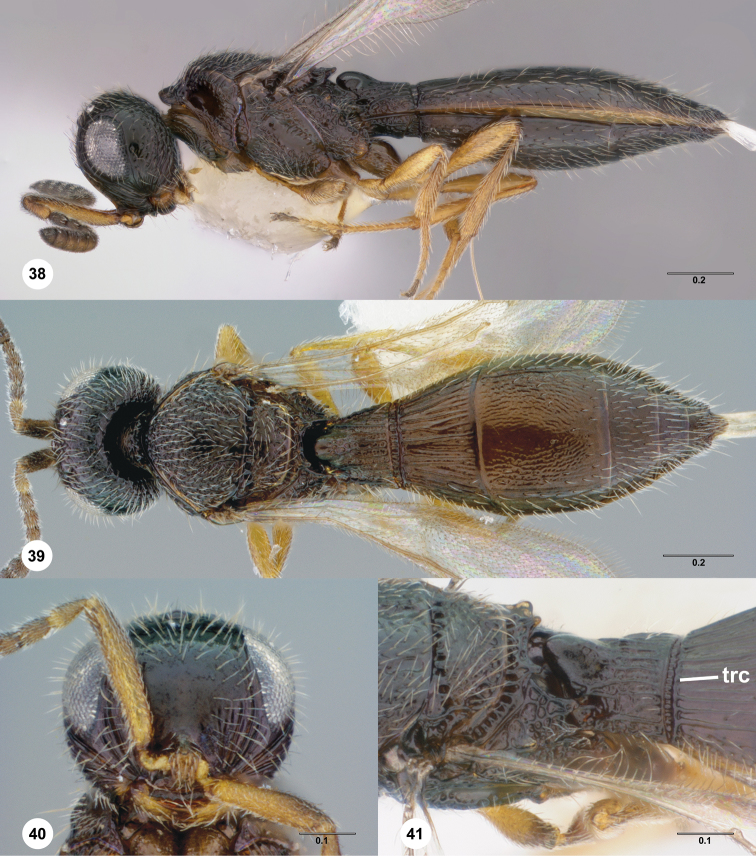
**^87^** *Paridris gorn*, sp. n. **38** Lateral habitus, female holotype (OSUC 405092) **39** Dorsal habitus, female (OSUC 334055) **40** Head, anterior view, female (OSUC 334054) **41** Mesoscutellum, metascutellum, propodeum, T1, T2, dorsolateral view, female (OSUC 405089).

### Paridris invicta

Talamas & Masner sp. n.

urn:lsid:zoobank.org:act: 107F45BB-5157-4A45-BFC9-A270F01C2089

urn:lsid:biosci.ohio-state.edu:osuc_concepts:298864

http://species-id.net/wiki/Paridris_invicta

Figures 11 42–45 Morphbank^29^

#### Description.

Male body length: 2.39 mm (n=1).

Color of head: black. Distal margin of clypeus: smooth. Width of clypeus: wider than interantennal process. Lateral corner of clypeus: projecting into acute angle. Development of interantennal process ventrally: not reaching clypeus. Number of mandibular teeth: three. Length of mediofacial striae: not extending above midpoint of eye. Shape of gena in dorsal view: moderately receding behind compound eye. Striae on gena: pronounced. Length of striae on gena: extending above ventral margin of eye. Distribution of microsculpture on head: absent. Length of OOL: less than 2 ocellar diameters. Occipital carina above foramen magnum: present. Anterior margin of occipital carina: crenulate. Setation of postgena: sparse. Ventral extent of occipital carina: extending to base of mandible.

Color of mesosoma: mesoscutellum brown, otherwise golden orange. Dorsal half of pronotal cervical sulcus: present as smooth furrow. Ventral half of pronotal cervical sulcus: present as line of small to minute cells. Transverse pronotal carina: present in posterior half of pronotum. Shape of pronotal shoulder in dorsal view: narrow and striplike. Form of pronotal suprahumeral sulcus: areolate. Macrosculpture of anterior medial mesoscutum: absent. Density of punctation on anterior medial mesoscutum: dense along mesoscutal suprahumeral sulcus, otherwise sparse. Reticulate microfissures on anterior half of medial mesoscutum: absent. Pustulate microsculpture on anterior mesoscutum: absent. Density of punctation on posterior medial mesoscutum: sparse. Notaulus: percurrent, reaching suprahumeral sulcus as a smooth furrow. Orientation of notauli: converging posteriorly. Shape of notaulus at posterior apex: ovoid. Macrosculpture of mesoscutellum: punctate rugose along margins, smooth medially. Postacetabular sulcus: crenulate. Mesopleural carina: absent. Punctures on posterodorsal mesepimeral area: absent. Sculpture of mesopleuron anteroventral to femoral depression: moderately punctate. Sculpture of posterior mesepimeral area: rugulose. Form of metascutellum in male: transverse lamella, posterior margin convex. Paracoxal and metapleural sulci: separate. Posterior margin of metapleuron below propodeal spiracle: with blunt angle near intersection with metapleural sulcus. Setation between metapleural triangle and metapleural sulcus: absent. Sculpture between metapleural triangle and metapleural sulcus: smooth. Sculpture of metapleural triangle: punctate rugose. Setation of metapleural triangle: moderately dense. Anterior propodeal projection: absent. Setation of metasomal depression: absent. Lateral propodeal area: raised above plical area and indicated by sparser setation. Plical carina: present. Shape of lateral propodeal area: separated from prespiracular propodeal area. Sculpture of lateral propodeal area: weakly to moderately rugose.

Color of metasoma: banded in pale and dark brown. Macrosculpture of T1: longitudinally strigose. Interstitial sculpture of T1: finely rugulose. Macrosculpture of T2 in male: longitudinally striate anteriorly, smooth posteriorly. Microsculpture on T2: absent. Setal patch of lateral T2: present throughout lateral surface of tergite. Posterior margin of transverse sulcus on T2: weakly convex. Carina along posterior margin of transverse sulcus on T2 in male: absent. Microsculpture on T3: absent. Macrosculpture of T3 medially in male: absent. Macrosculpture of T3 laterally in male: absent. Microsculpture on T4: absent. Macrosculpture of T4 in male: absent. Setation of S1: present as medial tuft. Form of S2 felt field: longitudinal row or patch of setigerous punctures. Macrosculpture of S2 medially: longitudinally striate. Macrosculpture of S3: absent.

Wing development: macropterous. Basal vein in hind wing: spectral. Setation of hind wing: reduced anad of submarginal vein. Length of postmarginalis: punctiform. RS+M in fore wing: spectral.

#### Diagnosis.

The form of the lateral propodeal area in *Paridris invicta* is unique among the New World species of *Paridris*. In members of the *Paridris pallipes* species group the plica is absent and thus there is no distinction between the plical area and lateral propodeal area. In other New World species the plica is well developed and the lateral propodeal area is contiguous with the prespiracular propodeal area. In *Paridris invicta*, the plica is distinct, and separates the plical area from the lateral propodeal area, and the lateral propodeal area is not contiguous with the prespiracular propodeal area; by this character alone it may be separated. In addition, the enlarged femora and dense, elongate setation of the head and mesosoma serve well to identify this species.

#### Etymology.

The Latin adjectival epithet “invicta” means “unconquered” or “strong”. It is given to this species for the large size of its legs and its powerful appearance.

#### Link to distribution map.

**^30^**

#### Material examined.

*Holotype*, male: **BRAZIL**: SP, Trilha da Anta, Base Barra Grande, MT B2, Intervales State Park, 13.XII–16.XII.2000, Malaise trap, M. T. Tavares et al., OSUC 236922 (deposited in MZSP).

**Figures 42–45.**
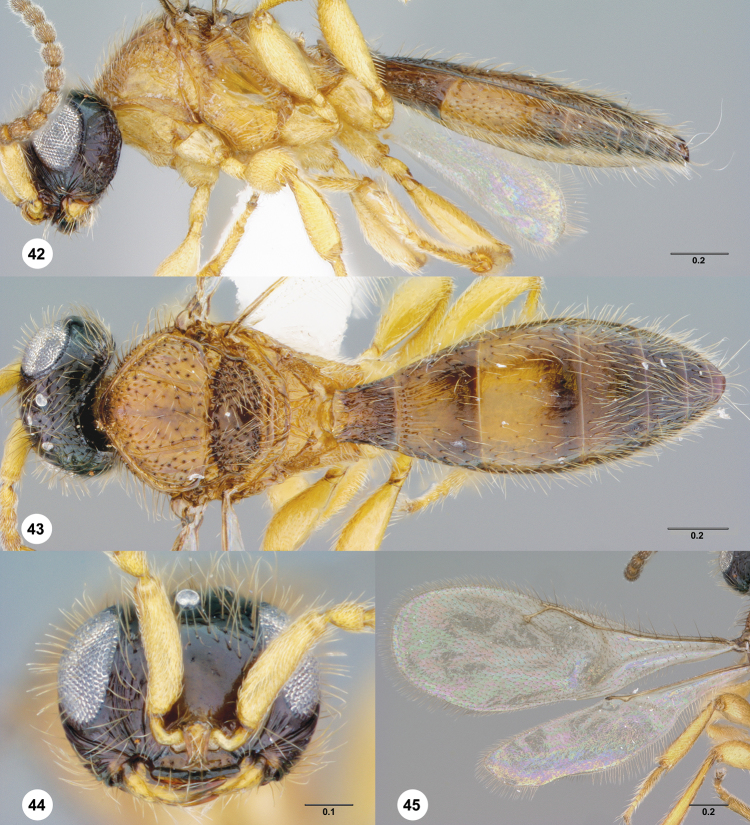
**^88^** *Paridris invicta*, sp. n. **42** Lateral habitus, male holotype (OSUC 236922) **43** Dorsal habitus, male holotype (OSUC 236922) **44** Head, anterior view, male holotype (OSUC 236922) **45** Wings, dorsal view, male holotype (OSUC 236922)

### Paridris isabelicae

Talamas & Masner sp. n.

urn:lsid:zoobank.org:act: 35C0E36A-79D9-4882-8B96-FDBD1E36E022

urn:lsid:biosci.ohio-state.edu:osuc_concepts:238223

http://species-id.net/wiki/Paridris_isabelicae

**Figures 46–49.**
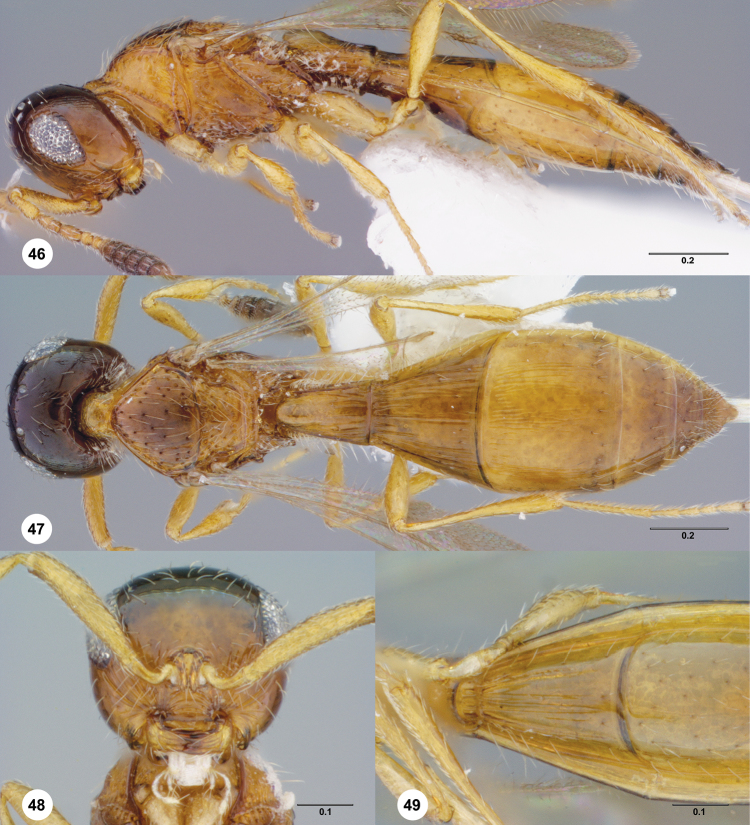
**^89^** *Paridris isabelicae*, sp. n. **46** Lateral habitus, female holotype (OSUC 334036) **47** Dorsal habitus, female (OSUC 334034) **48** Head, anterior view, female holotype (OSUC 334036) **49** S2, ventral view, male (OSUC 334044)

Figures 46–49 Morphbank^31^

#### Description.

Female body length: 1.43–1.99 mm (n=10). Male body length: 1.48–1.96 mm (n=18).

Number of basiconic sensilla on A8: 1.

Color of head: yellow; black; reddish brown. Distal margin of clypeus: smooth. Width of clypeus: wider than interantennal process. Lateral corner of clypeus: projecting into acute angle. Development of interantennal process ventrally: not reaching clypeus. Number of mandibular teeth: three. Length of mediofacial striae: not extending above midpoint of eye. Shape of gena in dorsal view: moderately receding behind compound eye. Striae on gena: weakly indicated. Length of striae on gena: terminating below ventral margin of eye. Form of microsculpture on head: reticulate microfissures. Distribution of microsculpture on head: present only on temples, between median and lateral ocellus, and posterior to lateral ocellus. Length of OOL: greater than 2 ocellar diameters. Occipital carina above foramen magnum: present. Anterior margin of occipital carina: simple. Setation of postgena: dense. Ventral extent of occipital carina: absent below midpoint of foramen magnum.

Color of mesosoma: yellow; reddish brown. Dorsal half of pronotal cervical sulcus: present as line of small to minute cells; present as line of large cells. Ventral half of pronotal cervical sulcus: present as line of large cells. Transverse pronotal carina: absent. Shape of pronotal shoulder in dorsal view: without dorsal surface. Form of pronotal suprahumeral sulcus: line of uniform punctures. Macrosculpture of anterior medial mesoscutum: absent. Density of punctation on anterior medial mesoscutum: sparse. Reticulate microfissures on anterior half of medial mesoscutum: absent. Pustulate microsculpture on anterior mesoscutum: absent. Density of punctation on posterior medial mesoscutum: sparse. Notaulus: percurrent, reaching suprahumeral sulcus as a smooth furrow. Orientation of notauli: parallel. Shape of notaulus at posterior apex: parallel-sided. Macrosculpture of mesoscutellum: absent. Postacetabular sulcus: crenulate. Mesopleural carina: present, complete. Punctures on posterodorsal mesepimeral area: absent. Sculpture of mesopleuron anteroventral to femoral depression: densely punctate; moderately punctate. Sculpture of posterior mesepimeral area: smooth. Form of metascutellum in female: transverse lamella, pointed medially. Form of metascutellum in male: transverse lamella, pointed medially; transverse lamella, posterior margin convex. Paracoxal and metapleural sulci: fused. Setation between metapleural triangle and metapleural sulcus: absent. Sculpture between metapleural triangle and metapleural sulcus: faintly rugulose. Sculpture of metapleural triangle: punctate rugose. Setation of metapleural triangle: sparse. Anterior propodeal projection: absent. Setation of metasomal depression: present. Lateral propodeal area: undifferentiated from plical area. Plical carina: absent.

Color of metasoma: yellow; reddish brown; yellowish brown. Macrosculpture of T1: longitudinally striate. Interstitial sculpture of T1: smooth. Adornment of horn on T1 in female: absent. Macrosculpture of T2 in female: longitudinally striate throughout. Macrosculpture of T2 in male: weakly longitudinally striate throughout. Microsculpture on T2: absent. Setal patch of lateral T2: present in thin line along lateral edge. Posterior margin of transverse sulcus on T2: distinctly convex. Carina along posterior margin of transverse sulcus on T2 in male: absent. Carina along posterior margin of transverse sulcus on T2 in female: absent. Microsculpture on T3: absent. Macrosculpture of T3 medially in female: longitudinally striate; absent. Macrosculpture of T3 laterally in female: longitudinally striate. Macrosculpture of T3 medially in male: weakly longitudinally striate. Macrosculpture of T3 laterally in male: weakly longitudinally striate. Microsculpture on T4: absent. Macrosculpture of T4 medially in female: absent. Macrosculpture of T4 laterally in female: absent. Macrosculpture of T4 in male: absent. Macrosculpture of T5 in female: absent. Constriction of apical T6 in female: present. Punctation of T6 in female: densely and finely punctate throughout. Setation of S1: densely present throughout. Form of S2 felt field: line of dense setae along longitudinal ridge. Macrosculpture of S2 medially: longitudinally striate. Macrosculpture of S3: absent.

Wing development: macropterous; brachypterous. Basal vein in hind wing: spectral. Setation of hind wing: reduced anad of submarginal vein. Length of postmarginalis: punctiform. RS+M in fore wing: nebulous.

#### Diagnosis.

*Paridris isabelicae* is similar to *Paridris convexa* and *Paridris saurotos*. Females of *Paridris saurotos* have a posteriorly projecting spine on the horn of T1, and those of *Paridris convexa* have a small transverse carina at the base of the horn. The horn of T1 is smooth in *Paridris isabelicae*. Males of *Paridris isabelicae* may be separated from the males of both other species by the presence of longitudinal striation on S2 medially.

#### Etymology.

This species is named for Cafetal La Isabelica, a coffee plantation where the holotype was collected. The epithet is treated as a noun in the genitive case.

#### Link to distribution map.

**^32^**

#### Material examined.

*Holotype*, female: **CUBA**: Santiago de Cuba Prov., La Isabelica, elfin forest, Gran Piedra Mountain, 1100m, 14.XII.1995, S. B. Peck, OSUC 334036 (deposited in CNCI). *Paratypes*: (23 females, 21 males) **CUBA**: 22 females, 16 males, OSUC 181295–181297, 334029, 334031–334032, 334034–334035, 334037, 334039, 334041–334047, 405059–405075 (CNCI); OSUC 334030, 334033, 334038, 334040 (OSUC). **DOMINICAN REPUBLIC**: 1 female, 5 males, OSUC 181344, 181384, 396496–396497, 396499 (CNCI); OSUC 396498 (OSUC).

### Paridris lemete

Talamas & Masner sp. n.

urn:lsid:zoobank.org:act: A382D0D8-F6F7-4BDD-A532-D5A2C31FBCEE

urn:lsid:biosci.ohio-state.edu:osuc_concepts:299225

http://species-id.net/wiki/Paridris_lemete

**Figures 50–53.**
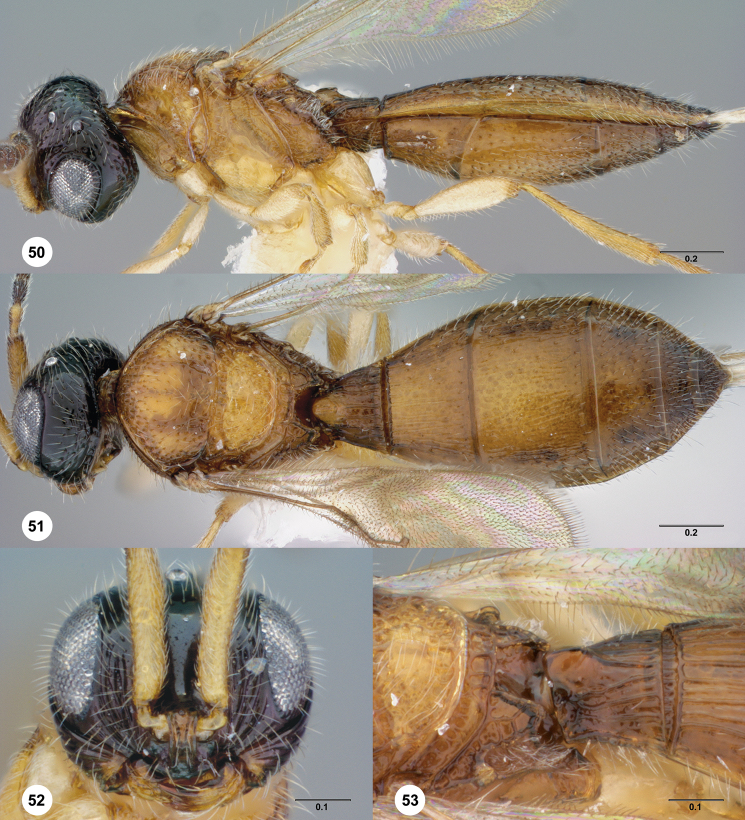
**^90^** *Paridris lemete*, sp. n. **50** Lateral habitus, female holotype (OSUC 334096) **51** Dorsal habitus, female holotype (OSUC 334096) **52** Head, anterior view, female holotype (OSUC 334096) **53** Mesoscutellum, metascutellum, propodeum, T1, T2, dorsolateral view, female (OSUC 334091)

Figures 50–53 86, 88 Morphbank^33^

#### Description.

Female body length: 2.16–2.35 mm (n=7). Male body length: 1.70–2.35 mm (n=20).

Number of basiconic sensilla on A8: 2.

Color of head: black; reddish brown. Distal margin of clypeus: serrate. Width of clypeus: equal to or less than width of interantennal process. Lateral corner of clypeus: projecting into acute angle. Development of interantennal process ventrally: connecting with clypeus. Number of mandibular teeth: three. Length of mediofacial striae: not extending above midpoint of eye. Shape of gena in dorsal view: moderately receding behind compound eye. Striae on gena: pronounced. Length of striae on gena: extending above ventral margin of eye. Distribution of microsculpture on head: absent. Length of OOL: less than 2 ocellar diameters. Occipital carina above foramen magnum: present. Anterior margin of occipital carina: comprised of small to miniscule cells. Setation of postgena: sparse. Ventral extent of occipital carina: extending to base of mandible.

Color of mesosoma: yellow; reddish brown; yellowish brown. Dorsal half of pronotal cervical sulcus: present as smooth furrow. Ventral half of pronotal cervical sulcus: present as line of small to minute cells. Transverse pronotal carina: present in posterior half of pronotum. Shape of pronotal shoulder in dorsal view: narrow and striplike. Form of pronotal suprahumeral sulcus: areolate. Macrosculpture of anterior medial mesoscutum: absent. Density of punctation on anterior medial mesoscutum: dense along mesoscutal suprahumeral sulcus, otherwise sparse. Reticulate microfissures on anterior half of medial mesoscutum: absent. Density of punctation on posterior medial mesoscutum: sparse. Notaulus: percurrent, reaching suprahumeral sulcus as a smooth furrow; percurrent, reaching suprahumeral sulcus as a line of punctures; abbreviate, not reaching mesoscutal suprahumeral sulcus. Orientation of notauli: parallel. Shape of notaulus at posterior apex: ovoid. Macrosculpture of mesoscutellum: punctate rugose along margins, smooth medially. Postacetabular sulcus: smoothly furrowed. Mesopleural carina: absent. Punctures on posterodorsal mesepimeral area: very fine; absent. Sculpture of mesopleuron anteroventral to femoral depression: smooth with punctures or rugulae along prespiracular sulcus. Sculpture of posterior mesepimeral area: smooth. Form of metascutellum in female: transverse punctate rugulose lamella, posterior margin approximately straight. Form of metascutellum in male: transverse punctate rugulose lamella, posterior margin approximately straight. Paracoxal and metapleural sulci: separate. Posterior margin of metapleuron below propodeal spiracle: with blunt angle near intersection with metapleural sulcus. Sculpture between metapleural triangle and metapleural sulcus: smooth. Sculpture of metapleural triangle: punctate rugose. Setation of metapleural triangle: sparse. Anterior propodeal projection: absent. Setation of metasomal depression: absent. Lateral propodeal area: raised above plical area and indicated by sparser setation. Plical carina: present. Shape of lateral propodeal area: continuous with prespiracular propodeal area. Sculpture of lateral propodeal area: rugose.

Color of metasoma: yellow; reddish brown; yellowish brown. Macrosculpture of T1: longitudinally striate. Interstitial sculpture of T1: smooth; finely rugulose. Adornment of horn on T1 in female: longitudinal median carina on dorsal surface, forming small point posteriorly. Macrosculpture of T2 in female: longitudinally striate throughout. Macrosculpture of T2 in male: longitudinally striate throughout. Microsculpture on T2: present. Setal patch of lateral T2: present throughout lateral surface of tergite. Posterior margin of transverse sulcus on T2: straight. Carina along posterior margin of transverse sulcus on T2 in male: present. Carina along posterior margin of transverse sulcus on T2 in female: present. Microsculpture on T3: present. Macrosculpture of T3 medially in female: weakly longitudinally striate. Macrosculpture of T3 laterally in female: longitudinally striate. Macrosculpture of T3 medially in male: weakly longitudinally striate. Macrosculpture of T3 laterally in male: longitudinally striate. Microsculpture on T4: absent. Macrosculpture of T4 medially in female: absent. Macrosculpture of T4 laterally in female: absent. Macrosculpture of T4 in male: absent. Macrosculpture of T5 in female: absent. Constriction of apical T6 in female: present. Punctation of T6 in female: densely and finely punctate throughout. Setation of S1: sparsely distributed throughout. Form of S2 felt field: longitudinal row or patch of setigerous punctures. Macrosculpture of S2 medially: longitudinally striate. Macrosculpture of S3: absent.

Wing development: macropterous. Basal vein in hind wing: spectral. Setation of hind wing: uniform throughout. Length of postmarginalis: approximately equal to length of stigmalis. RS+M in fore wing: spectral.

#### Diagnosis.

*Paridris lemete* is very similar to *Paridris aenea*. The additional basiconic sensillum present on A8 provides a straightforward character to separate the females of these species. Males of *Paridris lemete* are best separated from *Paridris aenea* by the smooth sculpture of the ventral mesopleuron.

#### Etymology.

The species epithet is derived from the Spanish phrase “le mete,” slang in Puerto Rico for “it is awesome”, which we which we consider to be appropriate for this species. The name is treated as a noun in apposition.

#### Link to distribution map.

**^34^**

#### Material examined.

*Holotype*, female: **PUERTO RICO**: Aguas Buenas Mpio., guano, Aguas Buenas Cave, 30.V.1974, Berlese funnel, S. Peck, OSUC 334096 (deposited in CNCI). *Paratypes*: **PUERTO RICO**: 6 females, 20 males, OSUC 181371, 334086–334089, 334091–334093, 334097, 396069, 396437–396442, 396444–396448 (CNCI); OSUC 334090, 334094–334095, 396443, 396449 (OSUC).

### Paridris minor

Talamas sp. n.

urn:lsid:zoobank.org:act: B6871632-ABE8-4476-94E2-0BFFACB1D30C

urn:lsid:biosci.ohio-state.edu:osuc_concepts:238224

http://species-id.net/wiki/Paridris_minor

**Figures 54–58.**
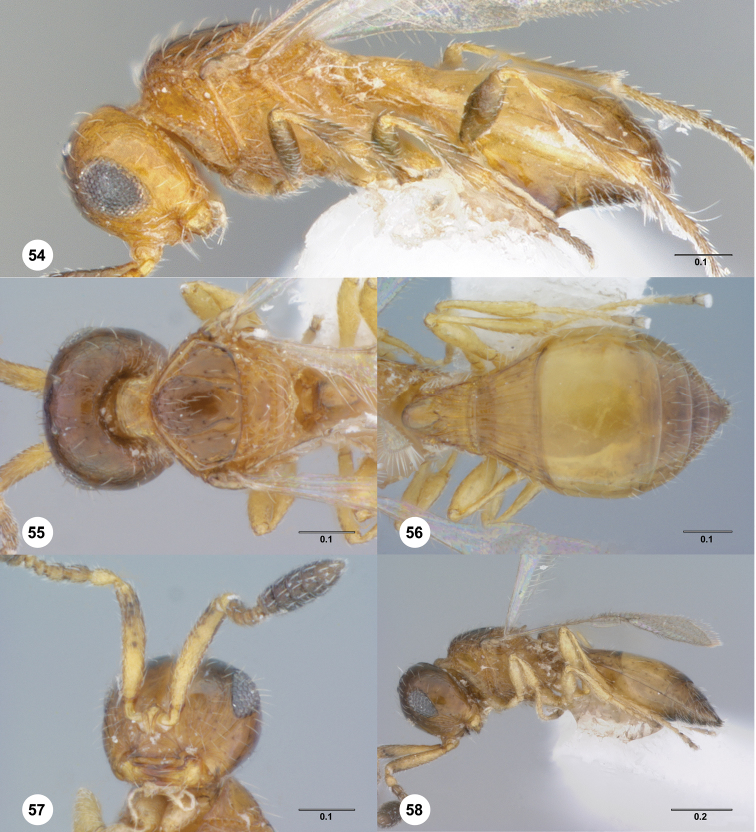
**^91^** *Paridris minor*, sp. n. **54** Lateral habitus, male (OSUC 265158) **55** Head and mesosoma, dorsal view, female holotype (OSUC 181299) **56** Metasoma, dorsal view, femalte holotype (OSUC 181299) **57** Head, anterolateral view, female holotype (OSUC 181299) **58** Lateral habitus, female holotype (OSUC 181299).

Figures 54–58 Morphbank^35^

#### Description.

Female body length: 1.11 mm (n=1). Male body length: 1.10 mm (n=1).

Number of basiconic sensilla on A8: 1.

Color of head: yellow; reddish brown. Distal margin of clypeus: smooth. Width of clypeus: wider than interantennal process. Lateral corner of clypeus: projecting into acute angle. Development of interantennal process ventrally: not reaching clypeus. Number of mandibular teeth: two. Length of mediofacial striae: not extending above midpoint of eye. Shape of gena in dorsal view: not receding or slightly bulging directly behind compound eye. Striae on gena: weakly indicated. Length of striae on gena: terminating below ventral margin of eye. Form of microsculpture on head: reticulate microfissures. Distribution of microsculpture on head: present throughout dorsal head. Length of OOL: greater than 2 ocellar diameters. Occipital carina above foramen magnum: present. Anterior margin of occipital carina: simple. Setation of postgena: dense. Ventral extent of occipital carina: absent below midpoint of foramen magnum.

Color of mesosoma: yellow. Dorsal half of pronotal cervical sulcus: present as line of small to minute cells. Ventral half of pronotal cervical sulcus: present as line of small to minute cells. Transverse pronotal carina: present in posterior half of pronotum; present in posterodorsal corner of pronotum. Shape of pronotal shoulder in dorsal view: narrow and striplike. Form of pronotal suprahumeral sulcus: broadly punctate. Macrosculpture of anterior medial mesoscutum: absent. Density of punctation on anterior medial mesoscutum: sparse. Reticulate microfissures on anterior half of medial mesoscutum: present throughout. Density of punctation on posterior medial mesoscutum: absent. Notaulus: percurrent, reaching suprahumeral sulcus as a smooth furrow. Orientation of notauli: converging posteriorly. Shape of notaulus at posterior apex: ovoid. Macrosculpture of mesoscutellum: absent. Postacetabular sulcus: crenulate. Mesopleural carina: absent. Punctures on posterodorsal mesepimeral area: absent. Sculpture of mesopleuron anteroventral to femoral depression: densely punctate. Sculpture of posterior mesepimeral area: smooth. Form of metascutellum in female: transverse lamella, pointed medially. Form of metascutellum in male: transverse lamella, pointed medially. Setation between metapleural triangle and metapleural sulcus: present throughout. Sculpture between metapleural triangle and metapleural sulcus: punctate. Sculpture of metapleural triangle: punctate rugose. Setation of metapleural triangle: sparse. Anterior propodeal projection: absent. Setation of metasomal depression: absent. Lateral propodeal area: undifferentiated from plical area. Plical carina: absent.

Color of metasoma: yellow anteriorly, brown posteriorly. Macrosculpture of T1: longitudinally striate. Adornment of horn on T1 in female: transverse ridge at base of horn. Macrosculpture of T2 in female: longitudinally striate throughout. Macrosculpture of T2 in male: longitudinally striate throughout. Microsculpture on T2: absent. Setal patch of lateral T2: present throughout lateral surface of tergite. Posterior margin of transverse sulcus on T2: distinctly convex. Carina along posterior margin of transverse sulcus on T2 in male: absent. Carina along posterior margin of transverse sulcus on T2 in female: absent. Microsculpture on T3: absent. Macrosculpture of T3 medially in female: absent. Macrosculpture of T3 laterally in female: absent. Macrosculpture of T3 medially in male: absent. Macrosculpture of T3 laterally in male: absent. Microsculpture on T4: absent. Macrosculpture of T4 medially in female: absent. Macrosculpture of T4 laterally in female: absent. Macrosculpture of T4 in male: absent. Macrosculpture of T5 in female: absent. Constriction of apical T6 in female: absent. Setation of S1: densely present throughout. Form of S2 felt field: line of dense setae along longitudinal ridge.

Wing development: macropterous. Basal vein in hind wing: spectral. Setation of hind wing: uniform throughout. RS+M in fore wing: nebulous.

#### Diagnosis.

*Paridris minor* shares with *Paridris convexa*, *Paridris gongylos*, and *Paridris laeviceps* the presence of reticulate microsculpture throughout the head. The females differ from *Paridris convexa* most notably by the shape of T6 which is not constricted in its apical half and from the females of *Paridris laeviceps* by the complete notaulus. Males of *Paridris minor* may be separated from these speces by the combination of the complete notaulus, a non-crenulate occipital rim and antennomeres 6–11 that are less than 3 times as long as wide.

#### Etymology.

This species is named for its diminutive size. The Latin epithet “minor” is treated as a noun in apposition.

#### Link to distribution map.

**^36^**

#### Material examined.

*Holotype*, female: **CUBA**: Santiago de Cuba Prov., botanical garden, Santiago de Cuba, 10m, 4.XII–17.XII.1995, yellow pan trap, L. Masner, OSUC 181298 (deposited in CNCI). *Paratype*: **CUBA**: 1 male, OSUC 265158 (CNCI).

### Paridris nayakorum

Talamas sp. n.

urn:lsid:zoobank.org:act: A72DF0F7-1FA3-4C3D-962A-071D91F6D894

urn:lsid:biosci.ohio-state.edu:osuc_concepts:299224

http://species-id.net/wiki/Paridris_nayakorum

Figures 6 59–62 84 Morphbank^37^

#### Description.

Female body length: 1.59–1.91 mm (n=19).

Number of basiconic sensilla on A8: 2.

Color of head: black. Distal margin of clypeus: serrate. Width of clypeus: wider than interantennal process. Lateral corner of clypeus: projecting into acute angle. Development of interantennal process ventrally: not reaching clypeus. Number of mandibular teeth: three. Length of mediofacial striae: not extending above midpoint of eye. Shape of gena in dorsal view: strongly receding behind compound eye. Striae on gena: weakly indicated. Length of striae on gena: terminating below ventral margin of eye. Form of microsculpture on head: reticulate microfissures. Distribution of microsculpture on head: present only on anterodorsal margin of eye, temples, and posterior to lateral ocellus. Length of OOL: greater than 2 ocellar diameters. Occipital carina above foramen magnum: present. Anterior margin of occipital carina: simple. Setation of postgena: dense. Ventral extent of occipital carina: absent below midpoint of foramen magnum.

Color of mesosoma: black. Dorsal half of pronotal cervical sulcus: present as line of large cells. Ventral half of pronotal cervical sulcus: present as line of large cells. Transverse pronotal carina: absent. Shape of pronotal shoulder in dorsal view: without dorsal surface. Form of pronotal suprahumeral sulcus: line of uniform punctures. Macrosculpture of anterior medial mesoscutum: absent. Density of punctation on anterior medial mesoscutum: moderate. Reticulate microfissures on anterior half of medial mesoscutum: present only along predicted notaular line. Pustulate microsculpture on anterior mesoscutum: absent. Density of punctation on posterior medial mesoscutum: moderately dense. Notaulus: present as single round depression at posterior margin of mesoscutum. Shape of notaulus at posterior apex: ovoid. Macrosculpture of mesoscutellum: absent. Postacetabular sulcus: comprised of distinct, closed cells. Mesopleural carina: absent. Punctures on posterodorsal mesepimeral area: absent. Sculpture of mesopleuron anteroventral to femoral depression: smooth. Sculpture of posterior mesepimeral area: smooth. Form of metascutellum in female: obscured by horn of T1. Paracoxal and metapleural sulci: fused. Setation between metapleural triangle and metapleural sulcus: present throughout. Sculpture between metapleural triangle and metapleural sulcus: punctate. Sculpture of metapleural triangle: densely punctate. Setation of metapleural triangle: dense. Anterior propodeal projection: present. Setation of metasomal depression: present. Lateral propodeal area: undifferentiated from plical area. Plical carina: absent.

Color of metasoma: brown; black. Macrosculpture of T1: absent. Adornment of horn on T1 in female: absent; longitudinal median carina at base of horn. Macrosculpture of T2 in female: striate anteriorly, with few striae reaching T3. Microsculpture on T2: absent. Setal patch of lateral T2: present in thin line along lateral edge. Posterior margin of transverse sulcus on T2: distinctly convex. Carina along posterior margin of transverse sulcus on T2 in female: absent. Microsculpture on T3: absent. Macrosculpture of T3 medially in female: absent. Macrosculpture of T3 laterally in female: absent. Microsculpture on T4: absent. Macrosculpture of T4 medially in female: absent. Macrosculpture of T4 laterally in female: absent. Macrosculpture of T5 in female: absent. Constriction of apical T6 in female: absent. Punctation of T6 in female: moderately dense along anterior margin. Setation of S1: densely present throughout. Form of S2 felt field: line of dense setae along longitudinal ridge. Macrosculpture of S2 medially: longitudinally striate. Macrosculpture of S3: absent.

Wing development: macropterous. Basal vein in hind wing: spectral. Setation of hind wing: uniform throughout. Length of postmarginalis: punctiform. RS+M in fore wing: nebulous.

#### Diagnosis.

*Paridris nayakorum* is most similar to *Paridris dnophos*, and may be separated easily by the presence of two basiconic sensilla on A8, the ovoid and abbreviate form of the notaulus, and the absence of longitudinal striae on T1. The large horn of T1 in *Paridris nayakorum* obscures the metascutellum in all specimens examined in this revision and is useful for separating it from most species of *Paridris*.

**Etymology**. *Paridris nayakorum* is named to commemorate the marriage of Dr. David A. Nayak (USA) and Alicia Rae Sim (USA), two friends of the first author.

#### Link to distribution map.

**^38^**

#### Material examined.

*Holotype*, female: **COSTA RICA**: Puntarenas Prov., Monteverde Cloud Forest Reserve, 25.V.1993, flight intercept trap, Michalski, OSUC 396697 (deposited in CNCI). *Paratypes*: **COSTA RICA**: 18 females, OSUC 262118–262121, 262128, 396349, 396352, 396356, 396358, 396362, 396677, 396680–396681, 396810 (CNCI); OSUC 262122, 396353, 396357, 396556 (OSUC).

#### Comments.

Although the males of the species are not yet known, we speculate that they will have a short ovoid notaulus and mesosomal sulci comprised of large cells, and that these characters will separate them from the males of *Paridris dnophos*.

**Figures 59–62.**
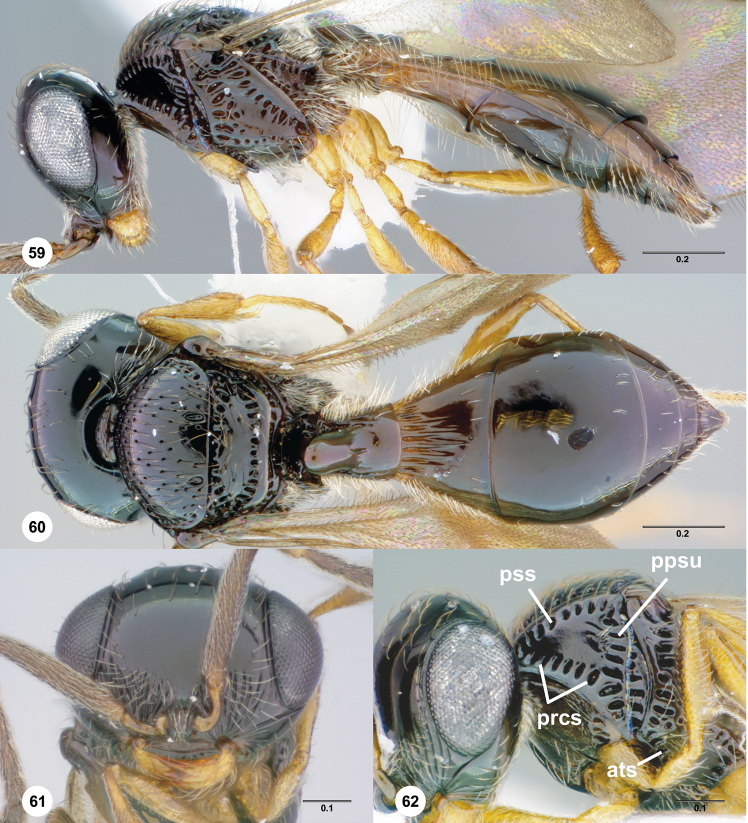
**^92^** *Paridris nayakorum*, sp. n. **59** Lateral habitus, female holotype (OSUC 396697) **60** Dorsal habitus, female (OSUC 396681) **61** Head, anterior view, female (OSUC 262128) **62** Pronotum, anterolateral view, female (OSUC 396358)

### Paridris pallipes

(Ashmead)

urn:lsid:zoobank.org:act:AF247306-37C6-41BA-B95A-DBF47D70E193

urn:lsid:biosci.ohio-state.edu:osuc_concepts:5079

http://species-id.net/wiki/Paridris_pallipes

Figures 2 63–66 83 Morphbank^39^

Thoron pallipes Ashmead, 1887: 99 (original description); Ashmead 1893: 168 (description); Kieffer 1926: 171, 173 (description, keyed).
Thoron pallidipes Ashmead: Dalla Torre 1898: 512 (emendation).
Paridris pallipes (Ashmead): Krombein and Burks 1967: 298 (generic transfer); Masner and Muesebeck 1968: 42 (type information). Morphbank^40^
Idris laeviceps Ashmead, 1893: 235 (original description), syn. n.
Idris leviceps Dalla Torre, 1898: 497 (unjustified emendation).
Paridris leviceps (Dalla Torre): Kieffer 1908: 123 (generic transfer).
Paridris laeviceps (Ashmead): Kieffer 1926: 422 (description, keyed); Masner and Muesebeck 1968: 42 (type information). urn:lsid:zoobank.org:act:2A5FC80F-CB07-4A53-B84A-0CA6D2DC53E7 urn:lsid:biosci.ohio-state.edu:osuc_concepts:5070 Morphbank^41^
Idris nigricornis Brues, 1903: 126 (original description), syn. n.; Brues 1916: 555 (description).
Paridris nigricornis (Brues): Kieffer 1908: 123 (generic transfer); Kieffer 1926: 422 (description, keyed); Masner 1965: 300 (lectotype designation). urn:lsid:zoobank.org:act:14146869-1734-402D-9215-58E8BF20FE64 urn:lsid:biosci.ohio-state.edu:osuc_concepts:5075 Morphbank^42^
Paridris brevipennis Fouts, 1920: 66 (original description), syn. n.; Masner and Muesebeck 1968: 42 (type information); Masner 1976: 36 (taxonomic status). urn:lsid:zoobank.org:act:B030A2A5-C38A-4EF8-9CD5-43835465F047 urn:lsid:biosci.ohio-state.edu:osuc_concepts:5064 Morphbank^43^

#### Description.

Female body length: 1.35–1.99 mm (n=20). Male body length: 1.32–1.95 mm (n=20).

Number of basiconic sensilla on A8: 1.

Color of head: brown; black; reddish brown. Distal margin of clypeus: serrate. Width of clypeus: wider than interantennal process. Lateral corner of clypeus: projecting into acute angle. Development of interantennal process ventrally: not reaching clypeus. Number of mandibular teeth: three. Length of mediofacial striae: not extending above midpoint of eye. Shape of gena in dorsal view: not receding or slightly bulging directly behind compound eye. Striae on gena: weakly indicated. Length of striae on gena: terminating below ventral margin of eye. Form of microsculpture on head: reticulate microfissures; pustulate. Distribution of microsculpture on head: present throughout dorsal head. Length of OOL: greater than 2 ocellar diameters; less than 2 ocellar diameters. Occipital carina above foramen magnum: present. Anterior margin of occipital carina: simple. Setation of postgena: dense. Ventral extent of occipital carina: absent below midpoint of foramen magnum.

Color of mesosoma: brown; black; reddish brown. Dorsal half of pronotal cervical sulcus: present as line of small to minute cells. Ventral half of pronotal cervical sulcus: present as line of large cells. Transverse pronotal carina: absent. Shape of pronotal shoulder in dorsal view: narrow and striplike. Form of pronotal suprahumeral sulcus: broadly punctate. Macrosculpture of anterior medial mesoscutum: absent. Density of punctation on anterior medial mesoscutum: dense along mesoscutal suprahumeral sulcus, otherwise sparse. Reticulate microfissures on anterior half of medial mesoscutum: present throughout. Density of punctation on posterior medial mesoscutum: dense; moderately dense. Notaulus: abbreviate, not reaching mesoscutal suprahumeral sulcus; present as single round depression at posterior margin of mesoscutum. Orientation of notauli: parallel. Shape of notaulus at posterior apex: ovoid. Macrosculpture of mesoscutellum: absent. Postacetabular sulcus: crenulate. Mesopleural carina: absent. Punctures on posterodorsal mesepimeral area: absent. Sculpture of mesopleuron anteroventral to femoral depression: densely punctate. Sculpture of posterior mesepimeral area: smooth. Form of metascutellum in female: transverse lamella, posterior margin convex. Form of metascutellum in male: transverse lamella, posterior margin convex. Paracoxal and metapleural sulci: fused. Posterior margin of metapleuron below propodeal spiracle: with blunt angle near intersection with metapleural sulcus. Setation between metapleural triangle and metapleural sulcus: present throughout. Sculpture between metapleural triangle and metapleural sulcus: punctate. Sculpture of metapleural triangle: densely punctate. Setation of metapleural triangle: dense. Anterior propodeal projection: absent. Setation of metasomal depression: absent. Lateral propodeal area: undifferentiated from plical area. Plical carina: absent.

Color of metasoma: brown; black; reddish brown. Macrosculpture of T1: longitudinally striate. Interstitial sculpture of T1: finely rugulose. Adornment of horn on T1 in female: absent. Macrosculpture of T2 in female: longitudinally striate throughout. Macrosculpture of T2 in male: longitudinally striate throughout. Microsculpture on T2: absent. Setal patch of lateral T2: present in thin line along lateral edge. Posterior margin of transverse sulcus on T2: distinctly convex. Carina along posterior margin of transverse sulcus on T2 in male: absent. Carina along posterior margin of transverse sulcus on T2 in female: absent. Microsculpture on T3: absent. Macrosculpture of T3 medially in female: absent. Macrosculpture of T3 laterally in female: absent. Macrosculpture of T3 medially in male: absent. Macrosculpture of T3 laterally in male: absent. Microsculpture on T4: absent. Macrosculpture of T4 medially in female: absent. Macrosculpture of T4 laterally in female: absent. Macrosculpture of T4 in male: absent. Macrosculpture of T5 in female: absent. Constriction of apical T6 in female: absent. Setation of S1: densely present throughout. Form of S2 felt field: line of dense setae along longitudinal ridge. Macrosculpture of S2 medially: longitudinally striate.

Wing development: macropterous; brachypterous. Basal vein in hind wing: spectral. Setation of hind wing: uniform throughout. RS+M in fore wing: nebulous.

#### Diagnosis.

Males of *Paridris pallipes* are similar to those of *Paridris gongylos* and may be easily separated by the simple occipital carina versus the crenulate occipital rim in *Paridris gongylos*. The dense microsculpture throughout the head and anterior mesosoma, absence of a transverse carina on T2 and smoothly convex posterior margin of T6 render the females of this species morphologically distinct among the specimens treated here.

#### Link to distribution map.

^44^

#### Associations.

collected on *Spartina alterniflora* Loisel.: [Cyperales: Poaceae]; collected on *alfalfa* : [Fabales: Fabaceae]; collected on *arroz* : [Cyperales: Poaceae]

#### Material examined.

*Holotype*, *Thoron pallipes*: **UNITED STATES**: Jacksnville, Fla; Type; type No. 24485 U.S.N.M.; Thoron pallipes Ashm. (USNM). *Holotype*, male, *Idris laeviceps*: **UNITED STATES**: VA, Arlington Co., Arlington, no date, USNM Type No. 24541 (deposited in USNM). *Lectotype*, *Idris nigricornis*: **UNITED STATES**: Mixed nest Myr-Lepto, Colebrook [CT], 9-10-01; LECTOTYPE Idris nigricornis Brues By L. Masner, 65; Idris nigricornis TYPES Brues; M.C.Z. type 31016 (MCZC). *Holotype*, *Paridris brevipennis*: **UNITED STATES**: Reared from eggs of Gryllus abbreviatus; Brookings S.D.; H.C. Severin Coll.; Type; Paridris brevipennis (MS) Fouts (USNM). *Other material*: (156 females, 153 males) **BELIZE**: 1 female, OSUC 181339 (CNCI). **BRAZIL**: 1 male, OSUC 323902 (OSUC). **CANADA**: 22 females, 25 males, OSUC 181096–181108, 181144, 181146, 181155–181156, 181167, 181169, 181175–181177, 334254–334258, 396139–396140, 396147–396158, 396183, 396235, 396240–396243 (CNCI). **COSTA RICA**: 3 males, OSUC 181333, 181395, 396126 (CNCI). **CUBA**: 1 female, 1 male, OSUC 436228–436229 (USNM). **GUATEMALA**: 1 female, OSUC 181365 (CNCI). **MEXICO**: 5 females, 2 males, OSUC 181180–181181, 181311, 181313, 396281, 396522 (CNCI); OSUC 436232 (USNM). **UNITED STATES**: 126 females, 121 males, OSUC 334293 (AMNH); CASENT 2042379–2042381, 2042383, 2042385, 2042387, 2042389–2042391 (CASC); OSUC 181109–181143, 181145, 181147, 181149–181154, 181157–181166, 181168, 181170–181174, 181178–181179, 181182–181185, 181279, 265156, 334259–334264, 396129–396138, 396141–396146, 396159–396182, 396184–396234, 396236–396239, 396270–396273, 396282–396297 (CNCI); OSUC 78732–78742 (MEMU); OSUC 141974, 176003, 207783, 254612, 256488, 256630–256631, 256784–256789, 266151–266155, 411762, 58699–58702 (OSUC); OSUC 205736 (UCDC); OSUC 157734, 157760 (UCMC); OSUC 436200–436206, 436208–436212 (USNM).

#### Comments.

*Paridris pallipes* exhibits remarkably little morphological variation for the large size of its geographical distribution. One specimen from Costa Rica, OSUC 265167, fits neatly into our concept of *Paridris pallipes* with the exception that it has a posteriorly directed spine on T1. Consequently, this specimen is determined only as *Paridris* until more specimens are available to assess if this is a morphological variation within *Paridris pallipes*, or if it should be treated as a separate species. The females of this species have macropterous and brachypterous forms. The lone specimen record of *Paridris pallipes* from Brazil (OSUC 323902) is worthy of mention because of its distance from any other specimen records, and may indicate that this species has been introduced to Brazil by humans.

**Figures 63–66.**
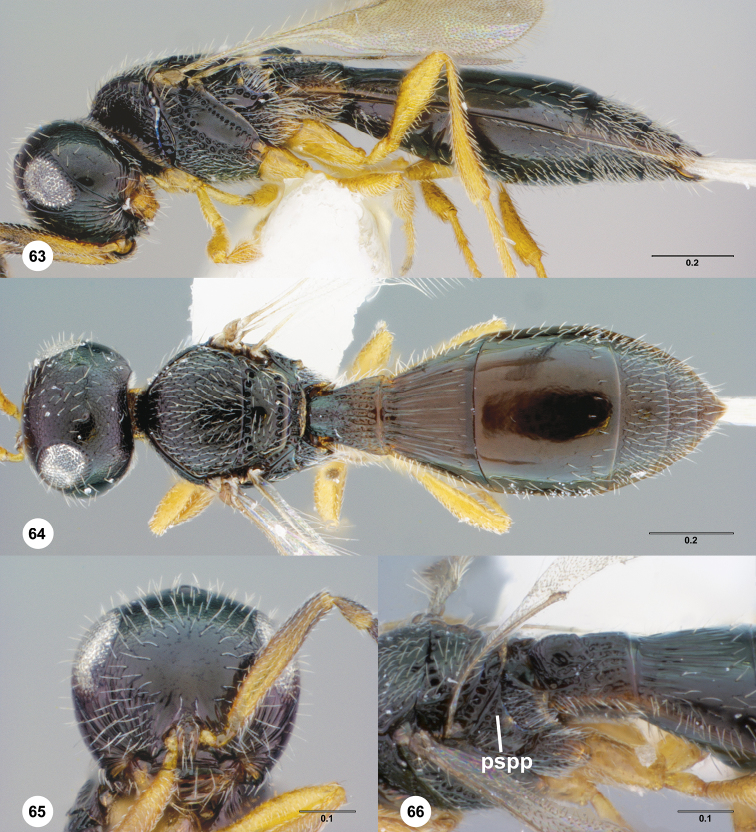
^93^ *Paridris pallipes* (Ashmead) **63** Lateral habitus, female (OSUC 256785) **64** Dorsal habitus, female (OSUC 396285) **65** Head, anterior view, female (OSUC 396295) **66** Mesocutellum, metanotum, propodeum, T1, T2, dorsolateral view, female (OSUC 207783)

### Paridris psydrax

Talamas & Masner sp. n.

urn:lsid:zoobank.org:act: F34200C7-2A71-4E1F-A363-D868DA380CEA

urn:lsid:biosci.ohio-state.edu:osuc_concepts:284314

http://species-id.net/wiki/Paridris_psydrax

Figures 5 67–70 80 Morphbank^45^

#### Description.

Female body length: 1.63–2.15 mm (n=5). Male body length: 1.79–1.93 mm (n=4).

Number of basiconic sensilla on A8: 1.

Color of head: brown; black; reddish brown. Distal margin of clypeus: serrate. Width of clypeus: wider than interantennal process. Lateral corner of clypeus: projecting into acute angle. Development of interantennal process ventrally: not reaching clypeus. Number of mandibular teeth: two; one. Length of mediofacial striae: not extending above midpoint of eye. Shape of gena in dorsal view: not receding or slightly bulging directly behind compound eye. Striae on gena: pronounced. Length of striae on gena: extending above ventral margin of eye. Form of microsculpture on head: pustulate. Distribution of microsculpture on head: present throughout dorsal head. Length of OOL: greater than 2 ocellar diameters. Occipital carina above foramen magnum: absent. Anterior margin of occipital carina: rounded. Setation of postgena: sparse. Ventral extent of occipital carina: extending to base of mandible.

Color of mesosoma: brown; black. Dorsal half of pronotal cervical sulcus: present as line of small to minute cells. Ventral half of pronotal cervical sulcus: present as line of small to minute cells. Transverse pronotal carina: present in posterior half of pronotum. Shape of pronotal shoulder in dorsal view: narrow and striplike. Form of pronotal suprahumeral sulcus: areolate; punctate rugulose. Macrosculpture of anterior medial mesoscutum: absent. Density of punctation on anterior medial mesoscutum: sparse. Reticulate microfissures on anterior half of medial mesoscutum: absent. Pustulate microsculpture on anterior mesoscutum: present. Density of punctation on posterior medial mesoscutum: sparse. Notaulus: percurrent, reaching suprahumeral sulcus as a line of punctures. Orientation of notauli: converging posteriorly. Shape of notaulus at posterior apex: ovoid. Macrosculpture of mesoscutellum: punctate rugose along margins, smooth medially. Postacetabular sulcus: crenulate. Mesopleural carina: present, complete. Punctures on posterodorsal mesepimeral area: absent. Sculpture of mesopleuron anteroventral to femoral depression: finely punctate. Sculpture of posterior mesepimeral area: smooth. Form of metascutellum in female: obscured by horn of T1. Form of metascutellum in male: transverse punctate rugulose lamella, posterior margin approximately straight. Paracoxal and metapleural sulci: separate. Posterior margin of metapleuron below propodeal spiracle: straight to moderately convex. Setation between metapleural triangle and metapleural sulcus: absent. Sculpture between metapleural triangle and metapleural sulcus: faintly rugulose. Sculpture of metapleural triangle: punctate rugose. Setation of metapleural triangle: sparse. Anterior propodeal projection: absent. Setation of metasomal depression: absent. Lateral propodeal area: raised above plical area and indicated by sparser setation. Plical carina: present. Shape of lateral propodeal area: continuous with prespiracular propodeal area. Sculpture of lateral propodeal area: punctate rugulose.

Color of metasoma: brown; reddish brown; yellowish brown. Macrosculpture of T1: rugose reticulate. Interstitial sculpture of T1: finely rugulose. Adornment of horn on T1 in female: absent. Macrosculpture of T2 in female: reticulate rugose throughout; reticulate; longitudinally strigose throughout. Macrosculpture of T2 in male: longitudinally strigose; weakly reticulate rugose. Microsculpture on T2: present. Setal patch of lateral T2: present throughout lateral surface of tergite. Posterior margin of transverse sulcus on T2: weakly convex. Carina along posterior margin of transverse sulcus on T2 in male: present. Carina along posterior margin of transverse sulcus on T2 in female: present. Microsculpture on T3: present. Macrosculpture of T3 medially in female: absent; reticulate. Macrosculpture of T3 laterally in female: absent; longitudinally strigose. Macrosculpture of T3 medially in male: absent. Macrosculpture of T3 laterally in male: weakly longitudinally striate; absent. Microsculpture on T4: absent. Macrosculpture of T4 medially in female: absent. Macrosculpture of T4 laterally in female: absent; longitudinally strigose. Macrosculpture of T4 in male: absent. Macrosculpture of T5 in female: absent. Constriction of apical T6 in female: present. Punctation of T6 in female: densely and finely punctate throughout; sparse along longitudinal midline and anterior margin, dense and fine laterally. Form of S2 felt field: longitudinal row or patch of setigerous punctures. Macrosculpture of S2 medially: crenulate. Macrosculpture of S3: absent.

Wing development: macropterous. Basal vein in hind wing: spectral. Setation of hind wing: reduced anad of submarginal vein. Length of postmarginalis: approximately half of length of stigmalis. RS+M in fore wing: spectral.

#### Diagnosis.

*Paridris psydrax* is a distinct species that is superficially similar to *Paridris pallipes* and *Paridris gongylos* in the dense microsculpture of the head and mesosoma. Females of *Paridris psydrax* may be identified by the large horn that obscures the metascutellum and the presence of a carina that posteriorly borders the transverse sulcus of T2. Males are best identified by the spherical shape of the antennal flagellomeres, the transverse carina on T2, and the presence of microsculpture on the head and mesosoma.

#### Etymology.

The Greek epithet psydrax, meaning “blister”, is given to the species for the pustulate microsculpture of the head and mesosoma. The name is treated as a noun in apposition.

#### Link to distribution map.

**^46^**

#### Material examined.

*Holotype*, female: **ARGENTINA**: Formosa Prov., 50km NW Clorinda, herbaceous vegetation, 90-121, Río Pilcomayo National Park, 19.XII.1990, sweeping, S. Peck & J. Peck, OSUC 181374 (deposited in CNCI). *Paratypes*: (7 females, 5 males) **MEXICO**: 1 male, OSUC 218772 (INHS). **PARAGUAY**: 3 females, OSUC 334217, 404962–404963 (OSUC). **UNITED STATES**: 3 females, 4 males, OSUC 181273–181274, 181276–181278 (CNCI); OSUC 181275 (LACM); OSUC 436207 (USNM). **VENEZUELA**: 1 female, OSUC 181378 (CNCI).

**Figures 67–70.**
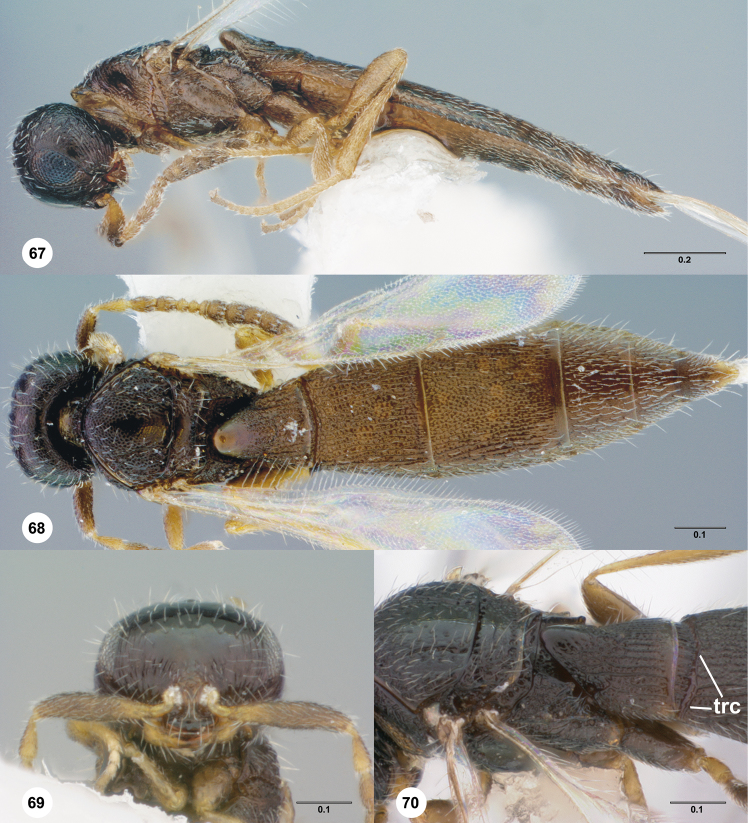
**^94^** *Paridris psydrax*, sp. n. **67** Lateral habitus, female (OSUC 181378) **68** Dorsal habitus, female holotype (OSUC 181374) **69** Head, anterior view, female holotype (OSUC 181374) **70** Mesoscutellum, metanotum, propodeum, T1, T2, dorsolateral view, female (OSUC 404963)

### Paridris saurotos

Talamas sp. n.

urn:lsid:zoobank.org:act: 1215ADEB-B74E-47BA-9463-2D58A3EDC8C2

urn:lsid:biosci.ohio-state.edu:osuc_concepts:299091

http://species-id.net/wiki/Paridris_saurotos

Figures 8 71–74 81 Morphbank^47^

#### Description.

Female body length: 2.00–2.88 mm (n=17). Male body length: 2.02–2.62 mm (n=10).

Number of basiconic sensilla on A8: 1.

Color of head: black; reddish brown. Distal margin of clypeus: smooth. Width of clypeus: wider than interantennal process. Lateral corner of clypeus: projecting into acute angle. Development of interantennal process ventrally: not reaching clypeus. Number of mandibular teeth: three. Length of mediofacial striae: not extending above midpoint of eye. Shape of gena in dorsal view: not receding or slightly bulging directly behind compound eye. Striae on gena: weakly indicated. Length of striae on gena: terminating below ventral margin of eye. Form of microsculpture on head: reticulate microfissures. Distribution of microsculpture on head: present only between median and lateral ocellus and on temples, in females present posterior to lateral ocellus. Length of OOL: greater than 2 ocellar diameters; less than 2 ocellar diameters. Occipital carina above foramen magnum: present. Anterior margin of occipital carina: simple. Setation of postgena: dense. Ventral extent of occipital carina: absent below midpoint of foramen magnum.

Color of mesosoma: reddish brown; yellowish brown. Dorsal half of pronotal cervical sulcus: present as line of small to minute cells. Ventral half of pronotal cervical sulcus: present as line of small to minute cells. Transverse pronotal carina: absent. Shape of pronotal shoulder in dorsal view: without dorsal surface. Form of pronotal suprahumeral sulcus: line of uniform punctures. Macrosculpture of anterior medial mesoscutum: absent. Density of punctation on anterior medial mesoscutum: dense along mesoscutal suprahumeral sulcus, otherwise sparse; moderate. Reticulate microfissures on anterior half of medial mesoscutum: present only along notaulus. Pustulate microsculpture on anterior mesoscutum: absent. Density of punctation on posterior medial mesoscutum: moderately dense. Notaulus: percurrent, reaching suprahumeral sulcus as a smooth furrow; percurrent, reaching suprahumeral sulcus as a line of punctures; abbreviate, not reaching mesoscutal suprahumeral sulcus. Orientation of notauli: parallel. Shape of notaulus at posterior apex: ovoid. Macrosculpture of mesoscutellum: punctate rugose. Postacetabular sulcus: crenulate. Mesopleural carina: present, complete. Punctures on posterodorsal mesepimeral area: absent. Sculpture of mesopleuron anteroventral to femoral depression: densely punctate; moderately punctate. Sculpture of posterior mesepimeral area: smooth. Form of metascutellum in female: transverse lamella, pointed medially. Form of metascutellum in male: transverse lamella, pointed medially. Paracoxal and metapleural sulci: fused. Posterior margin of metapleuron below propodeal spiracle: with blunt angle near intersection with metapleural sulcus. Setation between metapleural triangle and metapleural sulcus: absent. Sculpture between metapleural triangle and metapleural sulcus: smooth. Sculpture of metapleural triangle: finely punctate. Setation of metapleural triangle: moderately dense; sparse. Anterior propodeal projection: absent. Setation of metasomal depression: present. Lateral propodeal area: undifferentiated from plical area. Plical carina: absent.

Color of metasoma: yellow; reddish brown; yellowish brown. Macrosculpture of T1: longitudinally striate. Interstitial sculpture of T1: smooth. Adornment of horn on T1 in female: posteriorly projecting spine. Macrosculpture of T2 in female: longitudinally and sparsely striate, medial striae not reaching posterior margin. Macrosculpture of T2 in male: longitudinally and sparsely striate, medial striae not reaching posterior margin. Microsculpture on T2: absent. Setal patch of lateral T2: present in thin line along lateral edge. Carina along posterior margin of transverse sulcus on T2 in male: absent. Carina along posterior margin of transverse sulcus on T2 in female: absent. Microsculpture on T3: absent. Macrosculpture of T3 medially in female: absent. Macrosculpture of T3 laterally in female: weakly longitudinally striate; present as 1 or 2 strigae along junction of dorsal and lateral surfaces. Macrosculpture of T3 medially in male: absent. Macrosculpture of T3 laterally in male: weakly longitudinally striate; present as 1 or 2 strigae along junction of dorsal and lateral surfaces; absent. Microsculpture on T4: absent. Macrosculpture of T4 medially in female: absent. Macrosculpture of T4 laterally in female: absent. Macrosculpture of T4 in male: absent. Macrosculpture of T5 in female: absent. Constriction of apical T6 in female: present. Punctation of T6 in female: densely and finely punctate throughout. Setation of S1: densely present throughout. Form of S2 felt field: line of dense setae along longitudinal ridge. Macrosculpture of S2 medially: absent. Macrosculpture of S3: absent.

Wing development: macropterous; brachypterous. Basal vein in hind wing: spectral. Setation of hind wing: uniform throughout. Length of postmarginalis: less than half of length of stigmalis. RS+M in fore wing: nebulous.

#### Diagnosis.

*Paridris saurotos* is most similiar to *Paridris convexa* and *Paridris isabelicae* with which it shares elongate flagellomeres in males. The females may be quickly separated by the posteriorly directed spine on the horn of T1. Males of *Paridris saurotos* are best separated from *Paridris isabelicae* by the smooth sculpture of medial S2, which is longitudinally striate in the latter; and from *Paridris convexa* by the absence of microsculpture throughout the posterodorsal head.

#### Etymology.

The Greek “saurotos”, meaning “spiked”, refers to the posteriorly projecting spine on the horn of T1 in this species. The epithet is treated as a noun in apposition.

#### Link to distribution map.

**^48^**

#### Material examined.

*Holotype*, female: **JAMAICA**: Saint Andrew Parish, Hardwar Gap, 4000ft, 29.VII.1966, Howden & Becker, OSUC 262111 (deposited in CNCI). *Paratypes*:**JAMAICA**: 17 females, 10 males, OSUC 181357, 181373, 262106–262110, 262136, 265169–265172, 396245–396249, 396251–396253, 396256, 396268 (CNCI); OSUC 181394, 396244, 396250, 396254–396255 (OSUC).

**Figures 71–74.**
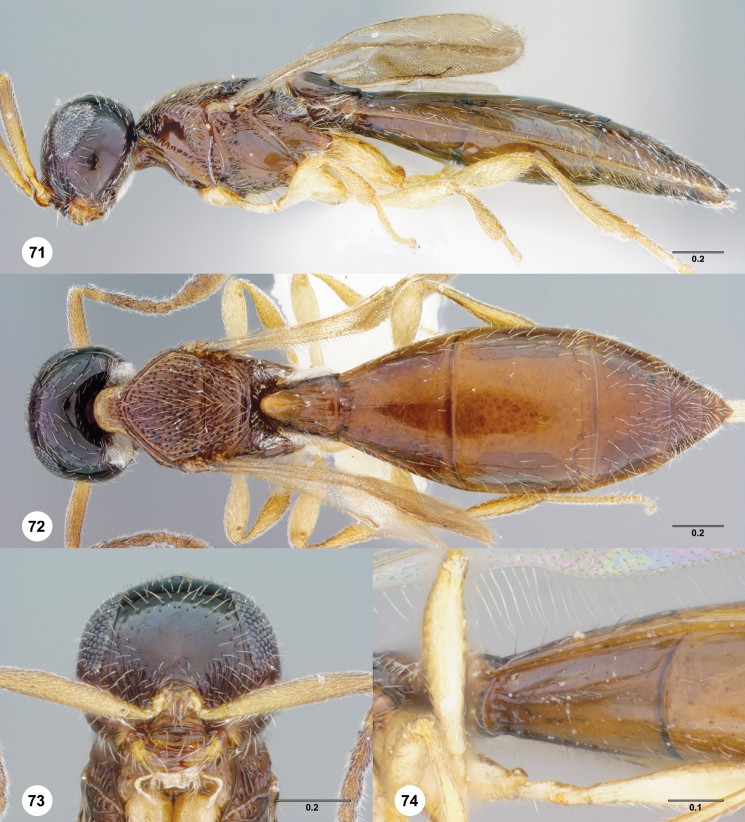
**^95^** *Paridris saurotos*, sp. n. **71** Lateral habitus, female holotype (OSUC 262111) **72** Dorsal haitus, female (OSUC 265171) **73** Head, anterior view, female (OSUC265171) **74** S2, ventrolateral view, female (OSUC 262107)

### Paridris soucouyant

Talamas & Masner sp. n.

urn:lsid:zoobank.org:act: 6EB3AE9A-9AC3-4C27-B60E-35B876FA91CD

urn:lsid:biosci.ohio-state.edu:osuc_concepts:298592

http://species-id.net/wiki/Paridris_soucouyant

**Figures 75–78.**
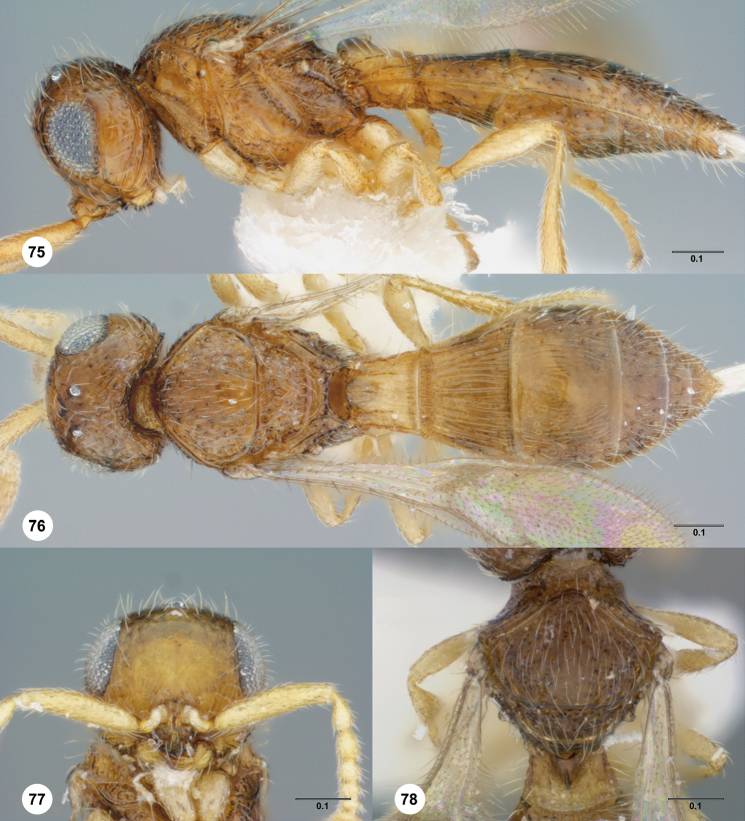
**^96^** *Paridris soucouyant*, sp. n. **75** Lateral habitus, female holotype (OSUC 396058) **76** Dorsal habitus, female holotype (OSUC 396058) **77** Head, anterior view, female (OSUC 396064) **78** Mesosoma and T1, dorsal view, female (OSUC 396064)

Figures 75–78 Morphbank^49^

#### Description.

Female body length: 1.36–1.49 mm (n=5).

Number of basiconic sensilla on A8: 1.

Color of head: reddish brown; yellowish brown. Distal margin of clypeus: serrate. Width of clypeus: equal to or less than width of interantennal process. Lateral corner of clypeus: rounded. Development of interantennal process ventrally: connecting with clypeus. Number of mandibular teeth: three. Length of mediofacial striae: not extending above midpoint of eye. Shape of gena in dorsal view: not receding or slightly bulging directly behind compound eye. Striae on gena: pronounced. Length of striae on gena: extending above ventral margin of eye. Distribution of microsculpture on head: absent. Length of OOL: greater than 2 ocellar diameters; less than 2 ocellar diameters. Occipital carina above foramen magnum: present. Anterior margin of occipital carina: rugose parallel to occipital carina; widely crenulate dorsally, smooth laterally. Setation of postgena: sparse. Ventral extent of occipital carina: extending to base of mandible.

Color of mesosoma: reddish brown; yellowish brown.Dorsal half of pronotal cervical sulcus: present as smooth furrow. Ventral half of pronotal cervical sulcus: present as line of small to minute cells. Transverse pronotal carina: present in posterior half of pronotum. Shape of pronotal shoulder in dorsal view: narrow and striplike. Form of pronotal suprahumeral sulcus: areolate. Macrosculpture of anterior medial mesoscutum: punctate rugose. Density of punctation on anterior medial mesoscutum: dense along mesoscutal suprahumeral sulcus, otherwise sparse. Reticulate microfissures on anterior half of medial mesoscutum: absent. Density of punctation on posterior medial mesoscutum: sparse. Notaulus: absent; abbreviate, not reaching mesoscutal suprahumeral sulcus. Orientation of notauli: parallel. Shape of notaulus at posterior apex: ovoid. Macrosculpture of mesoscutellum: punctate rugose. Postacetabular sulcus: crenulate. Mesopleural carina: present, complete. Punctures on posterodorsal mesepimeral area: absent. Sculpture of mesopleuron anteroventral to femoral depression: areolate to punctate rugose throughout. Sculpture of posterior mesepimeral area: smooth. Form of metascutellum in female: bispinose. Form of metascutellum in male: bispinose. Paracoxal and metapleural sulci: separate. Posterior margin of metapleuron below propodeal spiracle: straight to moderately convex. Setation between metapleural triangle and metapleural sulcus: absent. Sculpture between metapleural triangle and metapleural sulcus: punctate rugose. Sculpture of metapleural triangle: punctate rugose. Setation of metapleural triangle: sparse. Anterior propodeal projection: absent. Setation of metasomal depression: absent. Lateral propodeal area: raised above plical area and indicated by sparser setation. Plical carina: present. Shape of lateral propodeal area: connected to posteromedial corner of prespiracular propodeal area. Sculpture of lateral propodeal area: rugose.

Color of metasoma: yellowish brown; yellow anteriorly, brown posteriorly. Macrosculpture of T1: longitudinally striate. Interstitial sculpture of T1: finely rugulose. Adornment of horn on T1 in female: longitudinal median carina on dorsal surface, forming small point posteriorly. Macrosculpture of T2 in female: longitudinally striate throughout. Microsculpture on T2: absent. Setal patch of lateral T2: present throughout lateral surface of tergite. Posterior margin of transverse sulcus on T2: straight. Carina along posterior margin of transverse sulcus on T2 in female: present. Microsculpture on T3: present. Macrosculpture of T3 medially in female: absent. Macrosculpture of T3 laterally in female: longitudinally strigose. Microsculpture on T4: present. Macrosculpture of T4 medially in female: absent; rugulose. Macrosculpture of T4 laterally in female: rugulose; longitudinally strigose. Macrosculpture of T5 in female: absent along midline, rugulose laterally. Constriction of apical T6 in female: present. Punctation of T6 in female: densely and finely punctate throughout. Setation of S1: sparsely distributed throughout; absent. Macrosculpture of S2 medially: longitudinally striate. Macrosculpture of S3: absent.

Wing development: macropterous. Basal vein in hind wing: spectral. Setation of hind wing: uniform throughout. Length of postmarginalis: approximately equal to length of stigmalis. RS+M in fore wing: nebulous.

#### Diagnosis.

Among the species of the New World, *Paridris soucouyant* is most similar to *Paridris gorn*, with which it shares coarse punctation of the head and a characteristic shape of the metascutellum. The two are best separated by the presence of a longitudinal carina on the horn of T1 and rugulose sculpture of lateral T4–T5 in *Paridris soucouyant*. **Etymology**. This species is named for the soucouyant (pronounced sue-coo-yah) of Trinidadian folklore: a vampiric character that takes the form of a fireball and sucks the blood of its victims. The specific epithet is treated as a noun in apposition.

#### Link to distribution map.

**^50^**

#### Material examined.

*Holotype*, female: **TRINIDAD AND TOBAGO**: Tunapuna/Piarco Reg., Trinidad Isl., Santa Margarita Circular Road, Curepe, 13.VII–31.VIII.1974, E. D. Bennett, OSUC 396058 (deposited in CNCI). *Paratypes*: (4 females, 1 male) **COLOMBIA**: 1 male, OSUC 181401 (IAVH). **TRINIDAD AND TOBAGO**: 3 females, OSUC 396063–396065 (CNCI).**VENEZUELA**: 1 female, OSUC 181397 (CNCI).

**Figures 79–84.**
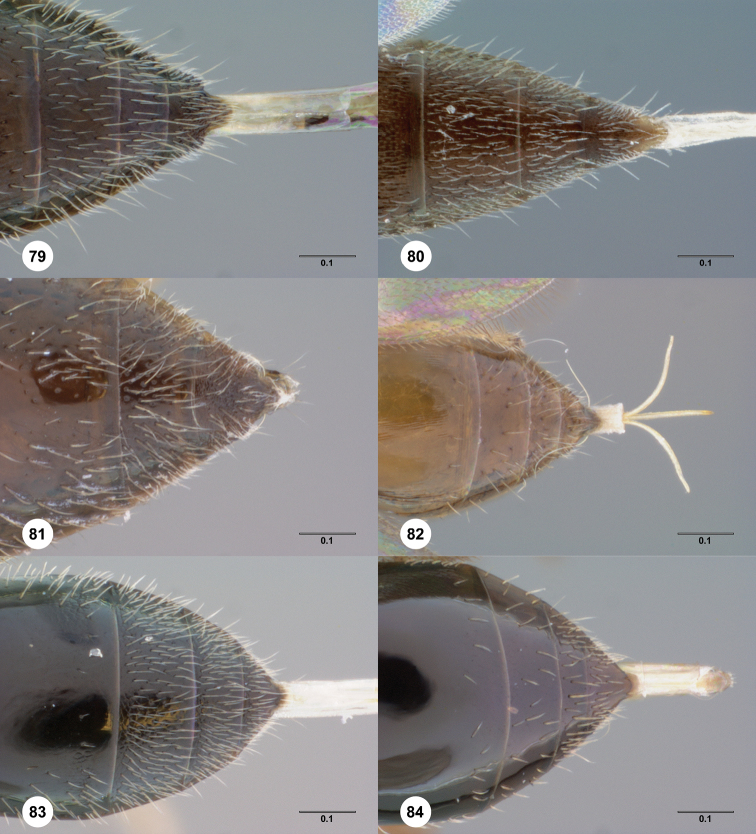
**^97^ 79** *Paridris gorn* sp. n.,T4–T6, dorsal view, female (OSUC 334054) **80** *Paridris psydrax* sp. n., T4–T6, dorsal view, female holotype (OSUC 181374) **81** *Paridris saurotos* sp. n., T5–T6, dorsal view, female holotype (OSUC 262111) **82** *Paridris convexa* sp. n., T4–T6, dorsal view, female holotype (OSUC 181392) **83** *Paridris pallipes* (Ashmead), T4–T6, dorsal view, female (OSUC 256785) **84** *Paridris nayakorum* sp. n., T4–T6, dorsal view, female (OSUC 262118)

**Figures 85–88.**
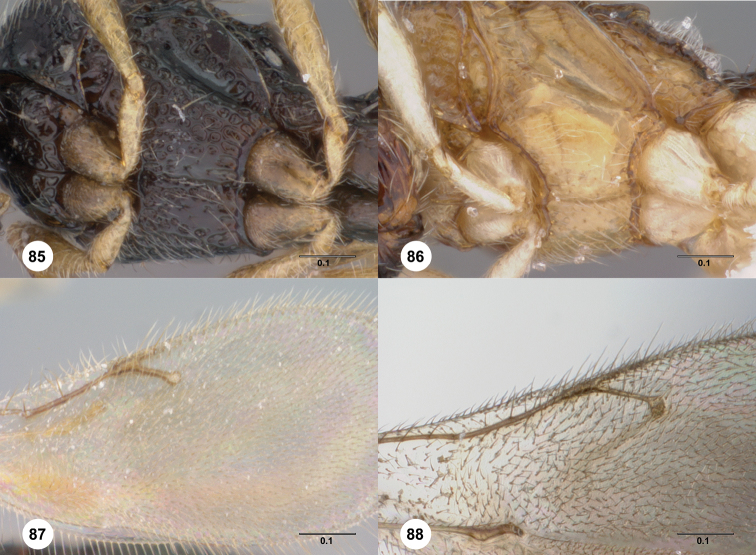
**^98^ 85** *Paridris aenea* (Ashmead), Mesopleuron, ventrolateral view, male (OSUC 396127) **86** *Paridris lemete* sp. n., Mesopleuron, ventrolateral view, male (OSUC 334089) **87** *Paridris aenea* (Ashmead), Venation of forewing, ventral view, female (OSUC 334201) **88** *Paridris lemete* sp. n., Venation of forewing, dorsal view, male (OSUC 334094)

### Probaryconus opacus

(Kieffer), comb. n.

http://species-id.net/wiki/Probaryconus_opacus

**Figures 89–94.**
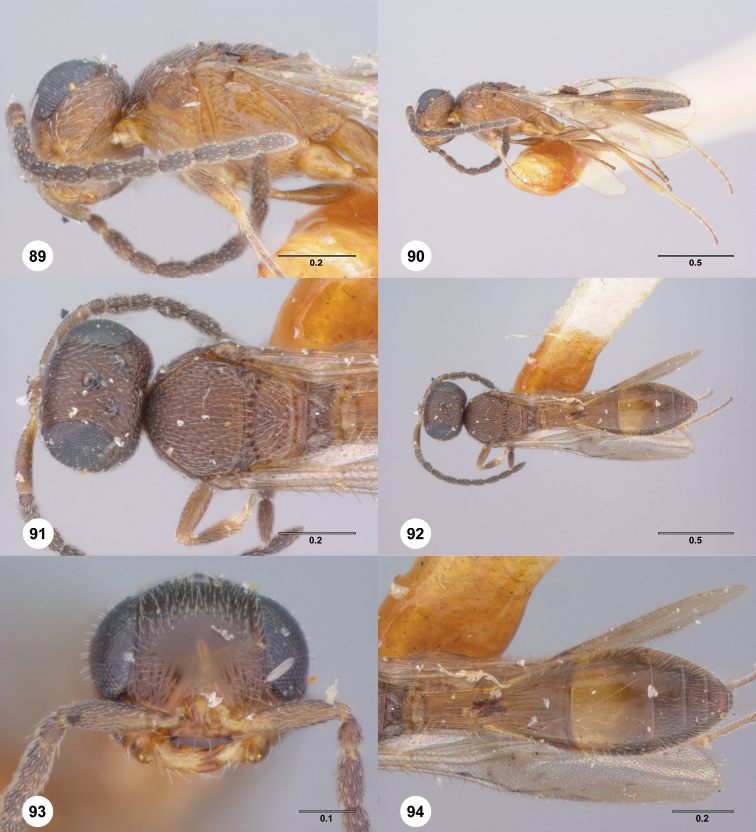
**^99^** *Probaryconus opacus* (Kieffer), male holotype (CAS Type No. 9711) **89** Head and mesosoma, lateral view **90** Lateral habitus **91** Head and mesosoma, dorsal view **92** Dorsal habitus **93** Head, anterior view **94** Metasoma, dorsal view

Figures 89–94 Morphbank^51^

Baryconus opacus Kieffer, 1910a: 320, 321 (original description, keyed).
Baryconus (Baryconus) opacus Kieffer: Kieffer 1910b: 84 (subgeneric assignment).
Paranteris opacus (Kieffer): Kieffer 1926: 430, 432 (generic transfer, description, keyed).
Paridris opaca (Kieffer): De Santis 1980: 314 (generic transfer). urn:lsid:zoobank.org:act: 86EFF18A-0F4A-4368-A11B-B7D9B39B747B urn:lsid:biosci.ohio-state.edu:osuc_concepts:5077

#### Comments.

Kieffer described *Baryconus opacus* from Brazil, and later transferred it to *Paranteris*, a genus synonymized with *Paridris* by Masner in 1965. Consistent with Kieffer’s interpretation of Foerster’s original concept of *Baryconus* (Ritchie and Masner 1983), *Paridris opaca* belongs in *Probaryconus*, a genus prone to confusion with *Paridris*.

## Supplementary Material

XML Treatment for Paridris aenea

XML Treatment for Paridris armata

XML Treatment for Paridris convexa

XML Treatment for Paridris dnophos

XML Treatment for Paridris gongylos

XML Treatment for Paridris gorn

XML Treatment for Paridris invicta

XML Treatment for Paridris isabelicae

XML Treatment for Paridris lemete

XML Treatment for Paridris minor

XML Treatment for Paridris nayakorum

XML Treatment for Paridris pallipes

XML Treatment for Paridris psydrax

XML Treatment for Paridris saurotos

XML Treatment for Paridris soucouyant

XML Treatment for Probaryconus opacus

## Endnotes

1 http://biocol.org:urn:lsid:biocol.org:col:34925

2 http://biocol.org:urn:lsid:biocol.org:col:1009

3 http://biocol.org:urn:lsid:biocol.org:col:1011

4 http://biocol.org:urn:lsid:biocol.org:col:1012

5 http://biocol.org/urn:lsid:biocol.org:col:1022

6 http://biocol.org/urn:lsid:biocol.org:col:1023

7 http://biocol.org/urn:lsid:biocol.org:col:34797

8 http://biocol.org/urn:lsid:biocol.org:col:34802

9 http://biocol.org/urn:lsid:biocol.org:col:33791

10 http://biocol.org:urn:lsid:biocol.org:col:33803

11 http://biocol.org:urn:lsid:biocol.org:col:33943

12 http://biocol.org/urn:lsid:biocol.org:col:33949

13 http://biocol.org:urn:lsid:biocol.org:col:1014

14 http://biocol.org:urn:lsid:biocol.org:col:34411

15 http://biocol.org:urn:lsid:biocol.org:col:34417

16 http://biocol.org/urn:lsid:biocol.org:col:1019

17 http://morphbank.net/?id=792538

18 http://hol.osu.edu/map-large.html?id=5062

19 http://morphbank.net/?id=792539

20 http://hol.osu.edu/map-large.html?id=298865

21 http://morphbank.net/?id=792541

22 http://hol.osu.edu/map-large.html?id=299093

23 http://morphbank.net/?id=792542

24 http://hol.osu.edu/map-large.html?id=299226

25 http://morphbank.net/?id=792543

26 http://hol.osu.edu/map-large.html?id=284313

27 http://morphbank.net/?id=792544

28 http://hol.osu.edu/map-large.html?id=299227

29 http://morphbank.net/?id=792545

30 http://hol.osu.edu/map-large.html?id=298864

31 http://morphbank.net/?id=792546

32 http://hol.osu.edu/map-large.html?id=238223

33 http://morphbank.net/?id=792548

34 http://hol.osu.edu/map-large.html?id=299225

35 http://morphbank.net/?id=792549

36 http://hol.osu.edu/map-large.html?id=238224

37 http://morphbank.net/?id=792550

38 http://hol.osu.edu/map-large.html?id=299224

39 http://morphbank.net/?id=792547

40 http://morphbank.net/?id=792594

41 http://morphbank.net/?id=792591

42 http://morphbank.net/?id=792590

43 http://morphbank.net/?id=792593

44 http://hol.osu.edu/map-large.html?id=5070

45 http://morphbank.net/?id=792551

46 http://hol.osu.edu/map-large.html?id=284314

47 http://morphbank.net/?id=792552

48 http://hol.osu.edu/map-large.html?id=299091

49 http://morphbank.net/?id=792553

50 http://hol.osu.edu/map-large.html?id=298592

51 http://morphbank.net/?id=792554

52 http://lsid.tdwg.org/urn:lsid:biosci.ohio-state.edu:osuc_pubs:68

53 http://lsid.tdwg.org/urn:lsid:biosci.ohio-state.edu:osuc_pubs:74

54 http://lsid.tdwg.org/urn:lsid:biosci.ohio-state.edu:osuc_pubs:75

55 http://lsid.tdwg.org/urn:lsid:biosci.ohio-state.edu:osuc_pubs:131

56 http://lsid.tdwg.org/urn:lsid:biosci.ohio-state.edu:osuc_pubs:167

57 http://lsid.tdwg.org/urn:lsid:biosci.ohio-state.edu:osuc_pubs:307

58 http://lsid.tdwg.org/urn:lsid:biosci.ohio-state.edu:osuc_pubs:235

59 http://lsid.tdwg.org/urn:lsid:biosci.ohio-state.edu:osuc_pubs:669

60 http://lsid.tdwg.org/urn:lsid:biosci.ohio-state.edu:osuc_pubs:339

61 http://lsid.tdwg.org/urn:lsid:biosci.ohio-state.edu:osuc_pubs:308

62 http://lsid.tdwg.org/urn:lsid:biosci.ohio-state.edu:osuc_pubs:400

63 http://lsid.tdwg.org/urn:lsid:biosci.ohio-state.edu:osuc_pubs:309

64 http://lsid.tdwg.org/urn:lsid:biosci.ohio-state.edu:osuc_pubs:310

65 http://lsid.tdwg.org/urn:lsid:biosci.ohio-state.edu:osuc_pubs:22163

66 http://lsid.tdwg.org/urn:lsid:biosci.ohio-state.edu:osuc_pubs:9730

67 http://lsid.tdwg.org/urn:lsid:biosci.ohio-state.edu:osuc_pubs:494

68 http://lsid.tdwg.org/urn:lsid:biosci.ohio-state.edu:osuc_pubs:9718

69 http://lsid.tdwg.org/urn:lsid:biosci.ohio-state.edu:osuc_pubs:236

70 http://lsid.tdwg.org/urn:lsid:biosci.ohio-state.edu:osuc_pubs:536

71 http://lsid.tdwg.org/urn:lsid:biosci.ohio-state.edu:osuc_pubs:311

72 http://lsid.tdwg.org/urn:lsid:biosci.ohio-state.edu:osuc_pubs:312

73 http://www.mapress.com/zootaxa/2007f/zt01571p078.pdf

74 http://lsid.tdwg.org/urn:lsid:biosci.ohio-state.edu:osuc_pubs:20959

75 http://lsid.tdwg.org/urn:lsid:biosci.ohio-state.edu:osuc_pubs:23517

76 http://lsid.tdwg.org/urn:lsid:biosci.ohio-state.edu:osuc_pubs:408

77 http://lsid.tdwg.org/urn:lsid:biosci.ohio-state.edu:osuc_pubs:23583

78 doi: 10.1371/journal.pone.0015991

79 http://morphbank.net/?id=792535

80 http://morphbank.net/?id=792536

81 http://morphbank.net/?id=792537

82 http://morphbank.net/?id=792555

83 http://morphbank.net/?id=792556

84 http://morphbank.net/?id=792557

85 http://morphbank.net/?id=792558

86 http://morphbank.net/?id=792559

87 http://morphbank.net/?id=792560

88 http://morphbank.net/?id=792561

89 http://morphbank.net/?id=792562

90 http://morphbank.net/?id=792564

91 http://morphbank.net/?id=792565

92 http://morphbank.net/?id=792566

93 http://morphbank.net/?id=792563

94 http://morphbank.net/?id=792567

95 http://morphbank.net/?id=792568

96 http://morphbank.net/?id=792569

97 http://morphbank.net/?id=792570

98 http://morphbank.net/?id=792571

99 http://morphbank.net/?id=792572

## Appendix 1

URI table of HAO morphological terms. (doi: 10.3897/zookeys.233.3455.app1) File format: Microsoft Word Open XML Document (DOCX).

**Copyright notice:** This dataset is made available under the Open Database License (http://opendatacommons.org/licenses/odbl/1.0/). The Open Database License (ODbL) is a license agreement intended to allow users to freely share, modify, and use this Dataset while maintaining this same freedom for others, provided that the original source and author(s) are credited.

## Appendix 2

Lucid key to species of *Paridris* in the New World. (doi: 10.3897/zookeys.233.3455.app2) File format: Lucid Key Data (lk4).

**Copyright notice:** This dataset is made available under the Open Database License (http://opendatacommons.org/licenses/odbl/1.0/). The Open Database License (ODbL) is a license agreement intended to allow users to freely share, modify, and use this Dataset while maintaining this same freedom for others, provided that the original source and author(s) are credited.
